# Supplementary material for: Ensemble-Based Virtual Screening Led to the Discovery of Novel Lead Molecules as Potential NMBAs
Source: Molecules. 2024 Apr 24;29(9):1955. doi: 10.3390/molecules29091955 (PMC11085220; doi:10.3390/molecules29091955)
Supplement: Supplementary file 1 [file molecules-29-01955-s001.zip › molecules-2948779-supplementary.pdf]

molecules

Supplementary Materials

Ensemble-Based Virtual Screening Led to the Discovery of Novel Lead Molecules as Potential NMBAs

Yi Zhang [1,2], Gonghui Ge [3], Xiangyang Xu [2,*] and Jinhui Wu [1,*]

1 School of Medicine, Nanjing University, Nanjing 210093, China
2 Jiangsu Key Laboratory of Central Nervous System Drug Research and Development, Jiangsu Nhwa Pharmaceutical Co., Ltd., Xuzhou 221116, China
3 School of Pharmacy, China Medical University, Shenyang 110122, China
* Correspondence: xuxiangyang@nhwa-group.com (X.X.); wuj@nju.edu.cn (J.W.)

Table S1. The NMBAs database collected from the literature and used in molecular property filters.

Compound	Formula	SMILES	DOI	M.W.	Charge	log*P*
**Patel1996-7**	C52H68N2O8	O=C(CC/C=C/CCC(OCCC[N+]1(C)CC(C=C(OC)C(OC)=C2)=C2C(CC3=CC=CC=C3)C1)=O)OCCC[N+]4(C)CC5=CC(OC)=C(OC)C=C5C(CC6=CC=CC=C6)C4	10.1016/0928-0987(95)00032-1	848	+2	4.036
**Patel1996-8**	C52H66F2N2O8	O=C(CC/C=C/CCC(OCCC[N+]1(C)CC(C=C(OC)C(OC)=C2)=C2C(CC3=CC(F)=CC=C3)C1)=O)OCCC[N+]4(C)CC5=CC(OC)=C(OC)C=C5C(CC6=CC(F)=CC=C6)C4	10.1016/0928-0987(95)00032-1	884	+2	4.322
**Patel1996-9**	C52H66Cl2N2O8	O=C(CC/C=C/CCC(OCCC[N+]1(C)CC(C=C(OC)C(OC)=C2)=C2C(CC3=CC=C(Cl)C=C3)C1)=O)OCCC[N+]4(C)CC5=CC(OC)=C(OC)C=C5C(CC6=CC=C(Cl)C=C6)C4	10.1016/0928-0987(95)00032-1	916	+2	5.462
**Patel1996-10**	C56H76N2O12	O=C(CC/C=C/CCC(OCCC[N+]1(C)CC(C=C(OC)C(OC)=C2)=C2C(CC3=CC(OC)=C(OC)C=C3)C1)=O)OCCC[N+]4(C)CC5=CC(OC)=C(OC)C=C5C(CC6=CC(OC)=C(OC)C=C6)C4	10.1016/0928-0987(95)00032-1	968	+2	3.352
**Brittain1977-1a**	C28H24N6	C[N+]1=C(N(/N=N/N2C(C=CC=C3)=C3[N+](C)=C2C4=CC=CC=C4)C5=C1C=CC=C5)C6=CC=CC=C6	10.1111/j.1476-5381.1977.tb09738.x	444	+2	-2.696
**Brittain1977-1b**	C30H28N6	C[N+]1=C(N(/N=N/N2C(C=CC=C3)=C3[N+](C)=C2C4=CC=C(C)C=C4)C5=C1C=CC=C5)C6=CC=C(C)C=C6	10.1111/j.1476-5381.1977.tb09738.x	472	+2	-1.698
**Brittain1977-1c**	C30H28N6O2	C[N+]1=C(N(/N=N/N2C(C=CC=C3)=C3[N+](C)=C2C4=CC=C(OC)C=C4)C5=C1C=CC=C5)C6=CC=C(OC)C=C6	10.1111/j.1476-5381.1977.tb09738.x	504	+2	-2.598
**Ginsburg1971-3**	C27H30NO2	O=C(OCC[N+](CC)(C1C(C=CC=C2)=C2C3=C1C=CC=C3)CC)CC4=CC=CC=C4	10.1111/j.1476-5381.1971.tb07161.x	400	+1	2.890
**Ginsburg1971-5**	C21H26N3O6	O=C(OCC[N+](CC)(CC1=CC=C([N+]([O-])=O)C=C1)CC)CC2=CC=C([N+]([O-])=O)C=C2	10.1111/j.1476-5381.1971.tb07161.x	416	+1	1.042
**Ginsburg1971-9**	C26H46N2O4	O=C(OCC[N+](CC)(CC)CC)CC1=C(CC(OCC[N+](CC)(CC)CC)=O)C=CC=C1	10.1111/j.1476-5381.1971.tb07161.x	450	+2	-2.712
**Ginsburg1971-11**	C26H46N2O4	O=C(OCC[N+](CC)(CC)CC)CC1=CC(CC(OCC[N+](CC)(CC)CC)=O)=CC=C1	10.1111/j.1476-5381.1971.tb07161.x	450	+2	-2.662
**Ginsburg1971-12**	C20H34N2O4	O=C(OCC[N+](C)(C)C)CC1=CC=C(CC(OCC[N+](C)(C)C)=O)C=C1	10.1111/j.1476-5381.1971.tb07161.x	366	+2	-5.836

**Ginsburg1971-13**	C26H46N2O4	O=C(OCC[N+](CC)(CC)CC)CC1=CC=C(CC(OCC[N+](CC)(CC)CC)=O)C=C1	10.1111/j.1476-5381.1971.tb07161.x	450	+2	-2.662
**Ginsburg1971-15**	C28H30NO2	O=C(OCC[N+](CC)(CC)C1C(C=CC=C2)=C2C3=C1C=CC=C3)/C=C/C4=CC=CC=C4	10.1111/j.1476-5381.1971.tb07161.x	412	+1	3.416
**Ginsburg1971-17**	C28H29ClNO2	O=C(OCC[N+](CC)(CC)C1C(C=CC=C2)=C2C3=C1C=CC=C3)/C=C/C4=CC=C(Cl)C=C4	10.1111/j.1476-5381.1971.tb07161.x	446	+1	4.139
**Ginsburg1971-19**	C28H29N2O4	O=C(OCC[N+](CC)(CC)C1C(C=CC=C2)=C2C3=C1C=CC=C3)/C=C/C4=C([N+]([O-])=O)C=CC=C4	10.1111/j.1476-5381.1971.tb07161.x	457	+1	3.169
**Ginsburg1971-21**	C28H29N2O4	O=C(OCC[N+](CC)(CC)C1C(C=CC=C2)=C2C3=C1C=CC=C3)/C=C/C4=CC([N+]([O-])=O)=CC=C4	10.1111/j.1476-5381.1971.tb07161.x	457	+1	3.169
**Ginsburg1971-23**	C28H29N2O4	O=C(OCC[N+](C1C(C=CC=C2)=C2C3=C1C=CC=C3)(CC)CC)/C=C/C4=CC=C([N+]([O-])=O)C=C4	10.1111/j.1476-5381.1971.tb07161.x	457	+1	3.169
**Ginsburg1971-25**	C28H32NO2	O=C(OCC[N+](CC)(C1C(C=CC=C2)=C2C3=C1C=CC=C3)CC)CCC4=CC=CC=C4	10.1111/j.1476-5381.1971.tb07161.x	414	+1	3.367
**Boros1999-1**	C54H71ClN2O14	C[N@@+]([C@@H]1CC2=CC(OC)=C(OC)C(OC)=C2)(CCCOC(/C=C(Cl)/C(OCCC[N@+]3(C)CCC(C=C(OC)C(OC)=C4)=C4[C@H]3CC5=CC(OC)=C(OC)C(OC)=C5)=O)=O)CCC6=C1C=C(OC)C(OC)=C6	10.1021/jm990060j	1006	+2	2.600
**Boros1999-2**	C52H67ClN2O14	C[N@@+](CCCOC(/C=C(C(OCCC[N@+]1(C)CCC2=C(C=C(C(OC)=C2)OC)[C@H]1C3=CC(OC)=C(OC)C(OC)=C3)=O)\Cl)=O)(CCC4=C5C=C(C(OC)=C4)OC)[C@H]5C6=CC(OC)=C(C(OC)=C6)OC	10.1021/jm990060j	978	+2	1.842
**Boros1999-3**	C52H68N2O14	C[N@@+](CCCOC(/C=C/C(OCCC[N@+]1(C)CCC2=C(C=C(C(OC)=C2)OC)[C@H]1C3=CC(OC)=C(OC)C(OC)=C3)=O)=O)(CCC4=C5C=C(C(OC)=C4)OC)[C@H]5C6=CC(OC)=C(C(OC)=C6)OC	10.1021/jm990060j	944	+2	1.189
**Boros1999-6a**	C53H69ClN2O14	C[N@+]1([C@@H](C(C=C2OC)=CC(OC)=C2OC)C(C(CC1)=C3)=CC(OC)=C3OC)CCCOC(/C=C(Cl)/C(OCCC[N@@+](CCC4=CC(OC)=C5OC)(C)[C@@H](C4=C5)CC(C=C6OC)=CC(OC)=C6OC)=O)=O	10.1021/jm990060j	992	+2	2.221
**Boros1999-4**	C52H67ClN2O14	C[N@+]([C@H]1C2=CC(OC)=C(OC)C(OC)=C2)(CCCOC(/C=C(Cl)/C(OCCC[N@@+]3(C)CCC(C=C(OC)C(OC)=C4)=C4[C@@H]3C5=CC(OC)=C(OC)C(OC)=C5)=O)=O)CCC6=C1C=C(OC)C(OC)=C6	10.1021/jm990060j	978	+2	1.842
**Laudexium**	C52H74N2O8	C[N+]1(CCCCCCCCCC[N+]2(C)C(CC3=CC=C(OC)C(OC)=C3)C(C=C(OC)C(OC)=C4)=C4CC2)C(CC5=CC(OC)=C(OC)C=C5)C6=CC(OC)=C(OC)C=C6CC1	10.1002/med.20036	854	+2	3.578
**Atracurium**	C53H72N2O12	C[N+]1(CCC(OCCCCCOC(CC[N+]2(C)C(CC3=CC=C(OC)C(OC)=C3)C(C=C(OC)C(OC)=C4)=C4CC2)=O)=O)C(CC5=CC(OC)=C(OC)C=C5)C6=CC(OC)=C(OC)C=C6CC1	10.1002/med.20036	928	+2	3.497
**Mivacurium**	C58H80N2O14	COC(C(OC)=C1)=C(OC)C=C1CC2[N+](C)(CCCOC(CC/C=C/CCC(OCCC[N+]3(C)C(CC4=CC(OC)=C(OC)C(OC)=C4)C5=CC(OC)=C(OC)C=C5CC3)=O)=O)CCC6=C2C=C(OC)C(OC)=C6	10.1002/med.20036	1028	+2	2.636
**Doxacurium**	C56H78N2O16	COC1=CC(CC2C3=C(C(OC)=C(C=C3CC[N+]2(CCCOC(CCC(OCCC[N+]4(CCC5=CC(OC)=C(C(OC)=C5C4CC6=CC(OC)=C(C(OC)=	10.1002/med.20036	1034	+2	1.042

		C6)OC)OC)C)=O)=O)C)OC)OC)=CC(OC)=C1OC				
**Oxalolaudonium**	C48H62N2O10	COC1=C(OC)C=C(C[N+](C)(CCCOC(C(OCCC[N+]2(C)C(CC3=CC=C(OC)C(OC)=C3)C(C=CC=C4)=C4CC2)=O)=O)C(CC5=CC=C(OC)C(OC)=C5)C6)C6=C1	10.1002/med.20036	826	+2	2.329
***d*-Tubocurarine**	C37H42N2O6	OC1=C(OC2=CC=C3C=C2)C([C@@]([N+](C)(C)CC4)([H])CC5=CC=C(O)C(OC(C(OC)=C6)=CC7=C6CC[N+](C)([H])[C@@]7([H])C3)=C5)=C4C=C1OC	10.1021/jm020574+	610	+2	4.412
**Metocurine**	C39H46N2O6	C[N+]1(C)[C@@](CC2=CC=C(O)C(OC(C(OC)=C3)=CC4=C3CC[N+](C)(C)[C@@]4([H])C5)=C2)([H])C6=C(CC1)C=C(OC)C(OC)=C6OC7=CC=C5C=C7	10.1021/jm020574+	638	+2	0.53
**Boros2003-20a**	C54H71ClN2O14	C[N+](CCC1=C2C=C(OC)C(OC)=C1)(CCCOC(/C(Cl)=C/C(OCCC[N+]3(C)C(CC4=CC(OC)=C(OC)C(OC)=C4)C(C=C(OC)C(OC)=C5)=C5CC3)=O)=O)C2CC6=CC(OC)=C(OC)C(OC)=C6	10.1021/jm020574+	1006	+2	2.600
**Boros2003-20b**	C52H63ClN2O14	C[N+](CCC1=C2C=C(OCO3)C3=C1)(CCCOC(/C(Cl)=C/C(OCCC[N+]4(C)C(CC5=CC(OC)=C(OC)C(OC)=C5)C(C=C(OCO6)C6=C7)=C7CC4)=O)=O)C2CC8=CC(OC)=C(OC)C(OC)=C8	10.1021/jm020574+	974	+2	3.214
**Boros2003-20c**	C52H67ClN2O14	C[N+]1(CCCOC(/C(Cl)=C/C(OCCC[N+]2(C)C(C3=CC(OC)=C(OC)C(OC)=C3)C(C=C(OC)C(OC)=C4)=C4CC2)=O)=O)C(C5=CC(OC)=C(OC)C(OC)=C5)C6=C(C=C(OC)C(OC)=C6)CC1	10.1021/jm020574+	978	+2	1.842
**Boros2003-21**	C52H68N2O14	C[N+]1(CCCOC(/C=C/C(OCCC[N+]2(C)C(C3=CC(OC)=C(OC)C(OC)=C3)C(C=C(OC)C(OC)=C4)=C4CC2)=O)=O)C(C5=CC(OC)=C(OC)C(OC)=C5)C6=C(C=C(OC)C(OC)=C6)CC1	10.1021/jm020574+	944	+2	1.189
**Boros2003-24g**	C54H71ClN2O15	COC1=C(OC)C([C@@H](CC2=CC(OC)=C(OC)C(OC)=C2)[N@@+](CCCOC(/C(Cl)=C/C(OCCC[N@@+]3(C)[C@@H](C4=CC(OC)=C(OC)C(OC)=C4)C(C=C(OC)C(OC)=C5)=C5CC3)=O)=O)(C)CC6)=C6C=C1OC	10.1021/jm020574+	1022	+2	1.863
**Boros2003-24i**	C52H67ClN2O14	COC1=CC([C@@H](CC2=CC(OC)=C(OC)C(OC)=C2)[N@@+](CCCOC(/C(Cl)=C/C(OCCC[N+]3([H])[C@@H](C4=CC(OC)=C(OC)C(OC)=C4)C(C=C(OC)C(OC)=C5)=C5CC3)=O)=O)(C)CC6)=C6C=C1OC	10.1021/jm020574+	978	+2	5.794
**Boros2003-24j**	C51H65ClN2O12	COC1=CC([C@@H](CC2=CC(OC)=C(OC)C(OC)=C2)[N@@+](CCCOC(/C(Cl)=C/C(OCCC[N@+]3(C)[C@@H](C4=CC=C(OC)C=C4)C(C=C(OC)C(OC)=C5)=C5CC3)=O)=O)(C)CC6)=C6C=C1OC	10.1021/jm020574+	932	+2	2.840
**Boros2003-24k**	C50H61ClF2N2O11	COC1=CC([C@@H](CC2=CC(OC)=C(OC)C(OC)=C2)[N@@+](CCCOC(/C(Cl)=C/C(OCCC[N@+]3(C)[C@@H](C4=CC=C(F)C(F)=C4)C(C=C(OC)C(OC)=C5)=C5CC3)=O)=O)(C)CC6)=C6C=C1OC	10.1021/jm020574+	938	+2	3.137
**Boros2003-24l**	C53H67ClN2O13	C[N+](CCC1=C2C=C(OC)C(OC)=C1)(CCCOC(/C(Cl)=C/C(OCCC[N+]34C(C5=CC(OC)=C(OC)C=C5CC4)C(C=C(OC)C(OC)=C6)=C6CC3)=O)=O)C2CC7=CC(OC)=C(OC)C(OC)=C7	10.1021/jm020574+	974	+2	2.444
**Boros2003-24m**	C53H67ClN2O13	C[N+](CCC1=C2C=C(OC)C(OC)=C1)(CCCOC(/C=C(Cl)/C(OCCC[N+]34C(C5=CC(OC)=C(OC)C=C5CC4)C(C=C(OC)C(OC)=C6)=C6CC3)=O)=O)C2CC7=CC(OC)=C(OC)C(OC)=C7	10.1021/jm020574+	974	+2	2.444

**Boros2003-24n**	C54H69ClN2O15	O=C(/C(Cl)=C/C(OCCC[N+]1(C)C(C2=CC(OC)=C(OC)C(OC)=C2)C(C=C(OC)C(OC)=C3)=C3CC1)=O)OCCC[N+]4(CCC5=C6C(OC)=C(OC)C(OC)=C5)C6CC7=C(C4)C(OC)=C(OC)C(OC)=C7	10.1021/jm020574+	1020	+2	1.519
**Boros2003-25a**	C57H80N2O14	C[N+](CCC1=C2C=C(OC)C(OC)=C1)(CCCOC(CCCCCCC(OCCC[N+]3(C)[C@H](C4=CC(OC)=C(OC)C(OC)=C4)C(C=C(OC)C(OC)=C5)=C5CC3)=O)=O)C2CC6=CC(OC)=C(OC)C(OC)=C6	10.1021/jm020574+	1016	+2	2.741
**Boros2003-25b**	C55H76N2O14	C[N+](CCC1=C2C=C(OC)C(OC)=C1)(CCCOC(CCCCC(OCCC[N+]3(C)[C@@H](C4=CC(OC)=C(OC)C(OC)=C4)C(C=C(OC)C(OC)=C5)=C5CC3)=O)=O)C2CC6=CC(OC)=C(OC)C(OC)=C6	10.1021/jm020574+	988	+2	1.683
**Boros2003-25c**	C56H78N2O14	C[N+](CCC1=C2C=C(OC)C(OC)=C1)(CCCOC(CCCCCC(OCCC[N+]3(C)[C@@H](C4=CC(OC)=C(OC)C(OC)=C4)C(C=C(OC)C(OC)=C5)=C5CC3)=O)=O)C2CC6=CC(OC)=C(OC)C(OC)=C6	10.1021/jm020574+	1002	+2	2.212
**Boros2003-25e**	C58H82N2O14	C[N+](CCC1=C2C=C(OC)C(OC)=C1)(CCCOC(CCCCCCCC(OCCC[N+]3(C)[C@@H](C4=CC(OC)=C(OC)C(OC)=C4)C(C=C(OC)C(OC)=C5)=C5CC3)=O)=O)C2CC6=CC(OC)=C(OC)C(OC)=C6	10.1021/jm020574+	1030	+2	3.270
**Boros2003-25f**	C59H84N2O14	C[N+](CCC1=C2C=C(OC)C(OC)=C1)(CCCOC(CCCCCCCCC(OCCC[N+]3(C)[C@@H](C4=CC(OC)=C(OC)C(OC)=C4)C(C=C(OC)C(OC)=C5)=C5CC3)=O)=O)C2CC6=CC(OC)=C(OC)C(OC)=C6	10.1021/jm020574+	1044	+2	3.799
**Boros2003-27a**	C53H71FN2O14	C[N+](CCC1=C2C=C(OC)C(OC)=C1)(CCCOC([C@@H](F)CC(OCCC[N+]3(C)[C@@H](C4=CC(OC)=C(OC)C(OC)=C4)C(C=C(OC)C(OC)=C5)=C5CC3)=O)=O)C2CC6=CC(OC)=C(OC)C(OC)=C6	10.1021/jm020574+	978	+2	1.502
**Boros2003-27b**	C54H72F2N2O14	C[N+](CCC1=C2C=C(OC)C(OC)=C1)(CCCOC(C(F)(CCC(OCCC[N+]3(C)[C@@H](C4=CC(OC)=C(OC)C(OC)=C4)C(C=C(OC)C(OC)=C5)=C5CC3)=O)F)=O)C2CC6=CC(OC)=C(OC)C(OC)=C6	10.1021/jm020574+	1010	+2	2.030
**Boros2003-27c**	C53H70F2N2O14	C[N+](CCC1=C2C=C(OC)C(OC)=C1)(CCCOC(C(CC(OCCC[N+]3(C)[C@@H](C4=CC(OC)=C(OC)C(OC)=C4)C(C=C(OC)C(OC)=C5)=C5CC3)=O)(F)F)=O)C2CC6=CC(OC)=C(OC)C(OC)=C6	10.1021/jm020574+	996	+2	2.575
**Boros2003-27d**	C54H72F2N2O15	C[N+](CCC1=C2C(OC)=C(OC)C(OC)=C1)(CCCOC(C(CC(OCCC[N+]3(C)[C@@H](C4=CC(OC)=C(OC)C(OC)=C4)C(C=C(OC)C(OC)=C5)=C5CC3)=O)(F)F)=O)C2CC6=CC(OC)=C(OC)C(OC)=C6	10.1021/jm020574+	1026	+2	2.217
**Boros2003-27f**	C53H70F2N2O14	C[N+](CCC1=C2C=C(OC)C(OC)=C1)(CCCOC(CC(F)(F)C(OCCC[N+]3(C)[C@@H](C4=CC(OC)=C(OC)C(OC)=C4)C(C=C(OC)C(OC)=C5)=C5CC3)=O)=O)C2CC6=CC(OC)=C(OC)C(OC)=C6	10.1021/jm020574+	996	+2	2.575
**Boros2003-28a**	C53H69FN2O14	C[N+](CCC1=C2C=C(OC)C(OC)=C1)(CCCOC(/C(F)=C/C(OCCC[N+]3(C)[C@@H](C4=CC(OC)=C(OC)C(OC)=C4)C(C=C(OC)C(OC)=C5)=C5CC3)=O)=O)C2CC6=CC(OC)=C(OC)C(OC)=C6	10.1021/jm020574+	976	+2	1.621
**Boros2003-28b**	C54H71FN2O15	C[N+](CCC1=C2C(OC)=C(OC)C(OC)=C1)(CCCOC(/C(F)=C/C(OCCC[N+]3(C)[C@@H](C4=CC(OC)=C(OC)C(OC)=C4)C(C=C(OC)C(OC)=C5)=C5CC3)=O)=O)C2CC6=CC(OC)=C(OC)C(OC)=C6	10.1021/jm020574+	1006	+2	1.263
**Gyermek2006-1**	C43H56N2O12	C[N@@+]1(CC2=CC(OC(C)=O)=C(OC(C)=O)C=C2)[C@H]3CCC[C@@H]1C[C@@H](OC(CC(O[C@H]4C[C@H]5CCC[C@@H](C4)[N@@+]5(CC6=CC=C(OC(C)=O)C(OC(C)=O)=C6)C)=O)=O)C3	10.1016/j.lfs.2006.01.0 38	792	+2	-4.445

**Gyermek2006-2**	C44H58N2O12	C[N@@+]1(CC2=CC(OC(C)=O)=C(OC(C)=O)C=C2)[C@H]3CCC[C@@H]1C[C@@H](OC(CCC(O[C@H]4C[C@H]5CCC[C@@H](C4)[N@@+]5(CC6=CC=C(OC(C)=O)C(OC(C)=O)=C6)C)=O)=O)C3	10.1016/j.lfs.2006.01.038	806	+2	-4.09
**Gyermek2006-3**	C45H60N2O12	C[N@@+]1(CC2=CC(OC(C)=O)=C(OC(C)=O)C=C2)[C@H]3CCC[C@@H]1C[C@@H](OC(CCCC(O[C@H]4C[C@H]5CCC[C@@H](C4)[N@@+]5(CC6=CC=C(OC(C)=O)C(OC(C)=O)=C6)C)=O)=O)C3	10.1016/j.lfs.2006.01.038	820	+2	-3.735
**Gyermek2006-4**	C46H62N2O12	C[N@@+]1(CC2=CC(OC(C)=O)=C(OC(C)=O)C=C2)[C@H]3CCC[C@@H]1C[C@@H](OC(CCCCC(O[C@H]4C[C@H]5CCC[C@@H](C4)[N@@+]5(CC6=CC=C(OC(C)=O)C(OC(C)=O)=C6)C)=O)=O)C3	10.1016/j.lfs.2006.01.038	834	+2	-3.786
**Gyermek2006-5**	C47H64N2O12	C[N@@+]1(CC2=CC(OC(C)=O)=C(OC(C)=O)C=C2)[C@H]3CCC[C@@H]1C[C@@H](OC(CCCCCC(O[C@H]4C[C@H]5CCC[C@@H](C4)[N@@+]5(CC6=CC=C(OC(C)=O)C(OC(C)=O)=C6)C)=O)=O)C3	10.1016/j.lfs.2006.01.038	848	+2	-3.257
**Gyermek2006-6**	C48H66N2O12	C[N@@+]1(CC2=CC(OC(C)=O)=C(OC(C)=O)C=C2)[C@H]3CCC[C@@H]1C[C@@H](OC(CCCCCCC(O[C@H]4C[C@H]5CCC[C@@H](C4)[N@@+]5(CC6=CC=C(OC(C)=O)C(OC(C)=O)=C6)C)=O)=O)C3	10.1016/j.lfs.2006.01.038	862	+2	-2.728
**Gyermek2006-7**	C44H56N2O12	C[N@@+]1(CC2=CC(OC(C)=O)=C(OC(C)=O)C=C2)[C@H]3CCC[C@@H]1C[C@@H](OC(/C=C/C(O[C@H]4C[C@H]5CCC[C@@H](C4)[N@@+]5(CC6=CC=C(OC(C)=O)C(OC(C)=O)=C6)C)=O)=O)C3	10.1016/j.lfs.2006.01.038	804	+2	-3.73
**Gyermek2006-8**	C44H55ClN2O12	C[N@@+]1(CC2=CC(OC(C)=O)=C(OC(C)=O)C=C2)[C@H]3CCC[C@@H]1C[C@@H](OC(/C(Cl)=C/C(O[C@H]4C[C@H]5CCC[C@@H](C4)[N@@+]5(CC6=CC=C(OC(C)=O)C(OC(C)=O)=C6)C)=O)=O)C3	10.1016/j.lfs.2006.01.038	838	+2	-3.08
**Gyermek2006-9**	C48H64N2O12	C[N@@+]1(CC2=CC(OC(C)=O)=C(OC(C)=O)C=C2)[C@H]3CCC[C@@H]1C[C@@H](OC(CC/C=C/CCC(O[C@H]4C[C@H]5CCC[C@@H](C4)[N@@+]5(CC6=CC=C(OC(C)=O)C(OC(C)=O)=C6)C)=O)=O)C3	10.1016/j.lfs.2006.01.038	860	+2	-3.212
**Gyermek2006-10**	C44H58N2O12S	C[N@@+]1(CC2=CC(OC(C)=O)=C(OC(C)=O)C=C2)[C@H]3CCC[C@@H]1C[C@@H](OC(CSCC(O[C@H]4C[C@H]5CCC[C@@H](C4)[N@@+]5(CC6=CC=C(OC(C)=O)C(OC(C)=O)=C6)C)=O)=O)C3	10.1016/j.lfs.2006.01.038	838	+2	-4.32
**Gyermek2006-11**	C46H60N2O12	C[N@@+]1(CC2=CC(OC(C)=O)=C(OC(C)=O)C=C2)[C@H]3CCC[C@@H]1C[C@@H](OC([C@@H]4[C@@H](CC4)C(O[C@H]5C[C@H]6CCC[C@@H](C5)[N@@+]6(CC7=CC=C(OC(C)=O)C(OC(C)=O)=C7)C)=O)=O)C3	10.1016/j.lfs.2006.01.038	832	+2	-3.806
**Gyermek2006-12**	C47H62N2O12	C[N@@+]1(CC2=CC(OC(C)=O)=C(OC(C)=O)C=C2)[C@H]3CCC[C@@H]1C[C@@H](OC([C@@H]4[C@@H](CCC4)C(O[C@H]5C[C@H]6CCC[C@@H](C5)[N@@+]6(CC7=CC=C(OC(C)=O)C(OC(	10.1016/j.lfs.2006.01.038	846	+2	-3.247

		C)=O)=C7)C)=O)=O)C3				
**Gyermek2006-13**	C48H64N2O12	C[N@@+]1(CC2=CC(OC(C)=O)=C(OC(C)=O)C=C2)[C@H]3CCC[C@@H]1C[C@@H](OC([C@@H]4[C@@H](CCCC4)C(O[C@H]5C[C@H]6CCC[C@@H](C5)[N@@+]6(CC7=CC=C(OC(C)=O)C(OC(C)=O)=C7)C)=O)=O)C3	10.1016/j.lfs.2006.01.038	860	+2	-2.688
**Gyermek2006-14**	C49H62N2O12	C[N@@+]1(CC2=CC(OC(C)=O)=C(OC(C)=O)C=C2)[C@H]3CCC[C@@H]1C[C@@H](OC(C4=C(C5CC[C@H]4C5)C(O[C@H]6C[C@H]7CCC[C@@H](C6)[N@@+]7(CC8=CC=C(OC(C)=O)C(OC(C)=O)=C8)C)=O)=O)C3	10.1016/j.lfs.2006.01.038	870	+2	-2.053
**Gyermek2006-15**	C48H66N2O12	C[N@@+]1(CC2=CC(OC(CC)=O)=C(OC(CC)=O)C=C2)[C@H]3CCC[C@@H]1C[C@@H](OC(CCC(O[C@H]4C[C@H]5CCC[C@@H](C4)[N@@+]5(CC6=CC=C(OC(CC)=O)C(OC(CC)=O)=C6)C)=O)=O)C3	10.1016/j.lfs.2006.01.038	862	+2	-1.974
**Gyermek2006-16**	C49H68N2O12	C[N@@+]1(CC2=CC(OC(CC)=O)=C(OC(CC)=O)C=C2)[C@H]3CCC[C@@H]1C[C@@H](OC(CCCC(O[C@H]4C[C@H]5CCC[C@@H](C4)[N@@+]5(CC6=CC=C(OC(CC)=O)C(OC(CC)=O)=C6)C)=O)=O)C3	10.1016/j.lfs.2006.01.038	876	+2	-1.619
**Gyermek2006-17**	C52H74N2O12	C[N@@+]1(CC2=CC(OC(CC)=O)=C(OC(CC)=O)C=C2)[C@H]3CCC[C@@H]1C[C@@H](OC(CCCCCCC(O[C@H]4C[C@H]5CCC[C@@H](C4)[N@@+]5(CC6=CC=C(OC(CC)=O)C(OC(CC)=O)=C6)C)=O)=O)C3	10.1016/j.lfs.2006.01.038	918	+2	-0.612
**Gyermek2006-18**	C48H64N2O12	C[N@@+]1(CC2=CC(OC(CC)=O)=C(OC(CC)=O)C=C2)[C@H]3CCC[C@@H]1C[C@@H](OC(/C=C/C(O[C@H]4C[C@H]5CCC[C@@H](C4)[N@@+]5(CC6=CC=C(OC(CC)=O)C(OC(CC)=O)=C6)C)=O)=O)C3	10.1016/j.lfs.2006.01.038	860	+2	-1.614
**Gyermek2006-19**	C48H63ClN2O12	C[N@@+]1(CC2=CC(OC(CC)=O)=C(OC(CC)=O)C=C2)[C@H]3CCC[C@@H]1C[C@@H](OC(/C(Cl)=C/C(O[C@H]4C[C@H]5CCC[C@@H](C4)[N@@+]5(CC6=CC=C(OC(CC)=O)C(OC(CC)=O)=C6)C)=O)=O)C3	10.1016/j.lfs.2006.01.038	894	+2	-0.961
**Gyermek2006-20**	C51H70N2O12	C[N@@+]1(CC2=CC(OC(CC)=O)=C(OC(CC)=O)C=C2)[C@H]3CCC[C@@H]1C[C@@H](OC([C@@H](CCC4)C4C(O[C@H]5C[C@H]6CCC[C@@H](C5)[N@@+]6(CC7=CC=C(OC(CC)=O)C(OC(CC)=O)=C7)C)=O)=O)C3	10.1016/j.lfs.2006.01.038	902	+2	-1.131
**Gyermek2006-21**	C52H72N2O12	C[N@@+]1(CC2=CC(OC(CC)=O)=C(OC(CC)=O)C=C2)[C@H]3CCC[C@@H]1C[C@@H](OC([C@@H](CCCC4)[C@@H]4C(O[C@H]5C[C@H]6CCC[C@@H](C5)[N@@+]6(CC7=CC=C(OC(CC)=O)C(OC(CC)=O)=C7)C)=O)=O)C3	10.1016/j.lfs.2006.01.038	916	+2	-0.572
**Gyermek2006-22**	C41H56N2O10	C[N@@+]1(CC2=CC(OC)=C(OC(C)=O)C=C2)[C@H]3CCC[C@@H]1C[C@@H](OC(CC(O[C@H]4C[C@H]5CCC[C@@H](C4)[N@@+]5(CC6=CC=C(OC(C)=O)C(OC)=C6)C)=O)=O)C3	10.1016/j.lfs.2006.01.038	736	+2	-3.305
**Gyermek2006-23**	C43H60N2O10	C[N@@+]1(CC2=CC(OC)=C(OC(C)=O)C=C2)[C@H]3CCC[C@@H]1C[C@@H](OC(CCCC(O[C@H]4C[C@H]5CCC[C@@H](C4)[N	10.1016/j.lfs.2006.01.038	764	+2	-2.595

		@@+]5(CC6=CC=C(OC(C)=O)C(OC)=C6)C)=O)=O)C3				
**Gyermek2006-24**	C45H64N2O10	C[N@@+]1(CC2=CC(OC)=C(OC(C)=O)C=C2)[C@H]3CCC[C@@H]1C[C@@H](OC(CCCCCC(O[C@H]4C[C@H]5CCC[C@@H](C4)[N@@+]5(CC6=CC=C(OC(C)=O)C(OC)=C6)C)=O)=O)C3	10.1016/j.lfs.2006.01.038	792	+2	-2.117
**Gyermek2006-25**	C46H66N2O10	C[N@@+]1(CC2=CC(OC)=C(OC(C)=O)C=C2)[C@H]3CCC[C@@H]1C[C@@H](OC(CCCCCCC(O[C@H]4C[C@H]5CCC[C@@H](C4)[N@@+]5(CC6=CC=C(OC(C)=O)C(OC)=C6)C)=O)=O)C3	10.1016/j.lfs.2006.01.038	806	+2	-1.588
**Gyermek2006-26**	C42H56N2O10	C[N@@+]1(CC2=CC(OC)=C(OC(C)=O)C=C2)[C@H]3CCC[C@@H]1C[C@@H](OC(/C=C/C(O[C@H]4C[C@H]5CCC[C@@H](C4)[N@@+]5(CC6=CC=C(OC(C)=O)C(OC)=C6)C)=O)=O)C3	10.1016/j.lfs.2006.01.038	748	+2	-2.590
**Gyermek2006-27**	C42H55ClN2O10	C[N@@+]1(CC2=CC(OC)=C(OC(C)=O)C=C2)[C@H]3CCC[C@@H]1C[C@@H](OC(/C(Cl)=C/C(O[C@H]4C[C@H]5CCC[C@@H](C4)[N@@+]5(CC6=CC=C(OC(C)=O)C(OC)=C6)C)=O)=O)C3	10.1016/j.lfs.2006.01.038	782	+2	-1.937
**Gyermek2006-28**	C42H58N2O10S	C[N@@+]1(CC2=CC(OC)=C(OC(C)=O)C=C2)[C@H]3CCC[C@@H]1C[C@@H](OC(CSCC(O[C@H]4C[C@H]5CCC[C@@H](C4)[N@@+]5(CC6=CC=C(OC(C)=O)C(OC)=C6)C)=O)=O)C3	10.1016/j.lfs.2006.01.038	782	+2	-3.180
**Gyermek2006-29**	C46H64N2O10	C[N@@+]1(CC2=CC(OC)=C(OC(C)=O)C=C2)[C@H]3CCC[C@@H]1C[C@@H](OC(C(CCCC4)[C@@H]4C(O[C@H]5C[C@H]6CCC[C@@H](C5)[N@@+]6(CC7=CC=C(OC(C)=O)C(OC)=C7)C)=O)=O)C3	10.1016/j.lfs.2006.01.038	804	+2	-1.548
**Gyermek2006-30**	C47H62N2O10	C[N@@+]1(CC2=CC(OC)=C(OC(C)=O)C=C2)[C@H]3CCC[C@@H]1C[C@@H](OC(C([C@H]4CC[C@H]5C4)=C5C(O[C@H]6C[C@H]7CCC[C@@H](C6)[N@@+]7(CC8=CC=C(OC(C)=O)C(OC)=C8)C)=O)=O)C3	10.1016/j.lfs.2006.01.038	814	+2	-0.913
**Gyermek2006-31**	C41H54Cl2N2O8	C[N@@+]1(CC2=CC(Cl)=C(OC(C)=O)C=C2)[C@H]3CCC[C@@H]1C[C@@H](OC(CCCC(O[C@H]4C[C@H]5CCC[C@@H](C4)[N@@+]5(CC6=CC=C(OC(C)=O)C(Cl)=C6)C)=O)=O)C3	10.1016/j.lfs.2006.01.038	772	+2	-0.827
**Gyermek2006-32**	C42H56Cl2N2O8	C[N@@+]1(CC2=CC(Cl)=C(OC(C)=O)C=C2)[C@H]3CCC[C@@H]1C[C@@H](OC(CCCCC(O[C@H]4C[C@H]5CCC[C@@H](C4)[N@@+]5(CC6=CC=C(OC(C)=O)C(Cl)=C6)C)=O)=O)C3	10.1016/j.lfs.2006.01.038	786	+2	-0.878
**Gyermek2006-33**	C41H54N4O12	C[N@@+]1(CC2=CC([N+]([O-])=O)=C(OC(C)=O)C=C2)[C@H]3CCC[C@@H]1C[C@@H](OC(CCCC(O[C@H]4C[C@H]5CCC[C@@H](C4)[N@@+]5(CC6=CC=C(OC(C)=O)C([N+]([O-])=O)=C6)C)=O)=O)C3	10.1016/j.lfs.2006.01.038	794	+2	-2.547
**Gyermek2006-34**	C45H62N2O8	C[N@@+]1(CC2=C(OC)C=CC(OC)=C2)[C@H]3CCC[C@@H]1C[C@@H](OC(C([C@H]4CC[C@H]5C4)=C5C(O[C@H]6C[C@H]7CCC[C@@H](C6)[N@@+]7(CC8=CC(OC)=CC=C8OC)C)=O)=O)C3	10.1016/j.lfs.2006.01.038	758	+2	0.927
**Gyermek2006-35**	C43H56N4O10	C[N@@+]1(CC2=C(OC)C=CC([N+]([O-])=O)=C2)[C@H]3CCC[C@@H]1C[C@@H](OC(C([C@H]4CC[C@H]5C4)=C5C(O[C@H]6C[C@H]7CCC[C@@H](C6)[N@@+]7(CC8=CC([N+]([O-])=O)=CC=C8OC)C)=O)=O)C3	10.1016/j.lfs.2006.01.038	788	+2	0.835
**Gyermek2006-36**	C45H64N2O12	C[N@@+]1(CC2=CC(OC)=C(OC(C)=O)C(OC)=C2)[C@H]3CCC[C	10.1016/j.lfs.2006.01.0	824	+2	-3.311

		@@H]1C[C@@H](OC(CCCC(O[C@H]4C[C@H]5CCC[C@@H](C4)[N@@+]5(CC6=CC(OC)=C(OC(C)=O)C(OC)=C6)C)=O)=O)C3	38			
**Gyermek2006-37**	C47H68N2O12	C[N@@+]1(CC2=CC(OC)=C(OC(C)=O)C(OC)=C2)[C@H]3CCC[C@@H]1C[C@@H](OC(CCCCCC(O[C@H]4C[C@H]5CCC[C@@H](C4)[N@@+]5(CC6=CC(OC)=C(OC(C)=O)C(OC)=C6)C)=O)=O)C3	10.1016/j.lfs.2006.01.038	852	+2	-2.833
**Gyermek2006-38**	C48H70N2O12	C[N@@+]1(CC2=CC(OC)=C(OC(C)=O)C(OC)=C2)[C@H]3CCC[C@@H]1C[C@@H](OC(CCCCCCC(O[C@H]4C[C@H]5CCC[C@@H](C4)[N@@+]5(CC6=CC(OC)=C(OC(C)=O)C(OC)=C6)C)=O)=O)C3	10.1016/j.lfs.2006.01.038	866	+2	-2.304
**Gyermek2006-39**	C44H62N2O12S	C[N@@+]1(CC2=CC(OC)=C(OC(C)=O)C(OC)=C2)[C@H]3CCC[C@@H]1C[C@@H](OC(CSCC(O[C@H]4C[C@H]5CCC[C@@H](C4)[N@@+]5(CC6=CC(OC)=C(OC(C)=O)C(OC)=C6)C)=O)=O)C3	10.1016/j.lfs.2006.01.038	842	+2	-3.896
**Gyermek2006-40**	C44H60N2O12	C[N@@+]1(CC2=CC(OC)=C(OC(C)=O)C(OC)=C2)[C@H]3CCC[C@@H]1C[C@@H](OC(/C=C/C(O[C@H]4C[C@H]5CCC[C@@H](C4)[N@@+]5(CC6=CC(OC)=C(OC(C)=O)C(OC)=C6)C)=O)=O)C3	10.1016/j.lfs.2006.01.038	808	+2	-3.306
**Gyermek2006-41**	C48H68N2O12	C[N@@+]1(CC2=CC(OC)=C(OC(C)=O)C(OC)=C2)[C@H]3CCC[C@@H]1C[C@@H](OC(C(CCCC4)[C@@H]4C(O[C@H]5C[C@H]6CCC[C@@H](C5)[N@@+]6(CC7=CC(OC)=C(OC(C)=O)C(OC)=C7)C)=O)=O)C3	10.1016/j.lfs.2006.01.038	864	+2	-2.264
**Gyermek2006-42**	C49H66N2O12	C[N@@+]1(CC2=CC(OC)=C(OC(C)=O)C(OC)=C2)[C@H]3CCC[C@@H]1C[C@@H](OC(C(C4CCC5C4)=C5C(O[C@H]6C[C@H]7CCC[C@@H](C6)[N@@+]7(CC8=CC(OC)=C(OC(C)=O)C(OC)=C8)C)=O)=O)C3	10.1016/j.lfs.2006.01.038	874	+2	-1.629
**Gyermek2006-43**	C47H68N2O12	C[N@@+]1(CC2=CC(OC)=C(OC(CC)=O)C(OC)=C2)[C@H]3CCC[C@@H]1C[C@@H](OC(CCCC(O[C@H]4C[C@H]5CCC[C@@H](C4)[N@@+]5(CC6=CC(OC)=C(OC(CC)=O)C(OC)=C6)C)=O)=O)C3	10.1016/j.lfs.2006.01.038	852	+2	-2.253
**Gyermek2006-44**	C49H64N2O16	C[N@@+]1(CC2=CC(OC(C)=O)=C(OC(C)=O)C(OC(C)=O)=C2)[C@H]3CCC[C@@H]1C[C@@H](OC(CCCC(O[C@H]4C[C@H]5CCC[C@@H](C4)[N@@+]5(CC6=CC(OC(C)=O)=C(OC(C)=O)C(OC(C)=O)=C6)C)=O)=O)C3	10.1016/j.lfs.2006.01.038	936	+2	-5.591
**Gyermek2006-45**	C49H62N2O16	C[N@@+]1(CC2=CC(OC(C)=O)=C(OC(C)=O)C(OC(C)=O)=C2)[C@H]3CCC[C@@H]1C[C@@H](OC(/C=C/CC(O[C@H]4C[C@H]5CCC[C@@H](C4)[N@@+]5(CC6=CC(OC(C)=O)=C(OC(C)=O)C(OC(C)=O)=C6)C)=O)=O)C3	10.1016/j.lfs.2006.01.038	934	+2	-5.619
**Booij2000-9c**	C14H30N4O2	C[N+](C)(C)CC(NCC(NCC[N+]1(CCCC1)C)=O)=O	10.1021/jm0010062	286	+2	-7.494
**Booij2000-11c**	C35H45N7O7	O=C(NCC(NCC(NCC[N+](CC1=CC=CC=C1)(CC2=CC=C([N+]([O-])=O)C=C2)C)=O)=O)C[N+]3(CC4=CC=C([N+]([O-])=O)C=C4)CCCCC3	10.1021/jm0010062	675	+2	-1.234
**Booij2000-15a**	C27H40N4O2	C[N+](CC1=CC=CC=C1)(CC2=CC=CC=C2)CC(NCC(NCC[N+]3(C)CCCCC3)=O)=O	10.1021/jm0010062	452	+2	-2.749

**Booij2000-15c**	C36H46N6O7	O=C(NC(CC1=CC=CC=C1)C(NCC[N+]2(CC3=CC=C([N+]([O-])=O)C=C3)CCOCC2)=O)C[N+]4(CC5=CC=C([N+]([O-])=O)C=C5)CCCCC4	10.1021/jm0010062	674	+2	1.416
**Booij2000-20a**	C26H45N5O3	C[N+]1(CC(N(C)CC(N(CC(N(CC[N+](C)(CC2=CC=CC=C2)C)C)=O)C)=O)=O)CCCCC1	10.1021/jm0010062	475	+2	-3.486
**Booij2000-20c**	C38H51N7O7	O=C(N(C)CC(N(CC(N(C)CC[N+](CC1=CC=C([N+]([O-])=O)C=C1)(C)CC2=CC=CC=C2)=O)C)=O)C[N+]3(CC4=CC=C([N+]([O-])=O)C=C4)CCCCC3	10.1021/jm0010062	717	+2	0.334
**Booij2000-20e**	C38H49N7O7	O=C(N(C)CC(N(CC(N1CC[N+](CC2=CC=CC=C2)(CC3=CC=C([N+]([O-])=O)C=C3)CC1)=O)C)=O)C[N+]4(CC5=CC=C([N+]([O-])=O)C=C5)CCCCC4	10.1021/jm0010062	715	+2	1.397
**Gyermek1999-1**	C43H54Br2N2O6	C[N@+]([C@](CC1)([H])C[C@H]2OC(CCC(O[C@@H](C[C@@]3([H])CC4)C[C@@]4([H])[N@+]3(C)/C=C/CC5=CC(C=CC(OC)=C6)=C6C(OC)=C5)=O)=O)([C@@]1([H])C2)CCC(C=C7)=CC(Br)=C7Br	WO9921854	852	+2	0.525
**Gyermek1999-2**	C42H58N2O12	C[N@@+]([C@H](CC1)CC2OC(CCC(OC(C[C@@H]3CC4)C[C@@H]4[N@@+]3(C)CC(C=C5OC)=CC(OC)=C5OC(C)=O)=O)=O)([C@@H]1C2)CC(C=C6OC)=CC(OC)=C6OC(C)=O	WO9921854	782	+2	-4.784
**Gyermek1999-3**	C50H70N2O12	C[N@@+]([C@H](CC1)CC2OC(CCC(OC(C[C@H]3CC4)C[C@H]4[N@+]3(C)CC(C=CC(OC(CCC)=O)=C5)=C5OC(CCC)=O)=O)=O)([C@@H]1C2)CC(C=CC(OC(CCC)=O)=C6)=C6OC(CCC)=O	WO9921854	890	+2	-0.276
**Gyermek1999-4**	C46H62N2O12	C[N@@+]([C@H](CC1)CC2OC(CCC(OC(C[C@H]3CC4)C[C@H]4[N@+]3(C)CC(C=C5)=CC(OC(CC)=O)=C5OC(CC)=O)=O)=O)([C@@H]1C2)CC(C=C6)=CC(OC(CC)=O)=C6OC(CC)=O	WO9921854	834	+2	-3.092
**Gyermek1999-5**	C36H48N4O10	C[N@@+]([C@H](CC1)CC2OC(CCC(OC(C[C@H]3CC4)C[C@H]4[N@+]3(C)CC(C=C5[N](=O)=O)=C(C=C5)OC)=O)=O)([C@@H]1C2)CC(C=C6[N](=O)=O)=C(C=C6)OC	WO9921854	696	+2	-2.32
**Gyermek1999-6**	C42H58N2O12	C[N@@+]([C@H](CC1)CC2OC(CCC(OC(C[C@@H]3CC4)C[C@@H]4[N@@+]3(C)CC(C=C5OC)=CC(OC)=C5OC(C)=O)=O)=O)([C@@H]1C2)CC(C=C6OC)=CC(OC)=C6OC(C)=O	WO9921854	782	+2	-4.784
**Gyermek1999-7**	C38H48Cl2N2O8	C[N@@+]([C@H](CC1)CC2OC(CCC(OC(C[C@H]3CC4)C[C@H]4[N@+]3(C)CC(C=C5)=CC(Cl)=C5OC(C)=O)=O)=O)([C@@H]1C2)CC(C=C6)=CC(Cl)=C6OC(C)=O	WO9921854	730	+2	-2.30
**Gyermek1999-8**	C44H58N2O12	C[N+](C(CC1)CC2OC(CCC(OC(CC3CC4)CC4[N+]3(C)CCC(C=C5)=CC(OC(C)=O)=C5OC(C)=O)=O)=O)(C1C2)CCC(C=C6)=CC(OC(C)=O)=C6OC(C)=O	WO9921854	806	+2	-6.25
**Gyermek1999-9**	C42H54N2O12	C[N@+]([C@@H](CC1)CC2OC(CCC(OC(C[C@H]3CC4)C[C@H]4[N@+]3(C)CC(C=C5OC(C)=O)=C(C=C5)OC(C)=O)=O)=O)([C@H]1C2)CC(C=C6OC(C)=O)=C(C=C6)OC(C)=O	WO9921854	778	+2	-4.508
**Gyermek1999-10**	C40H54N2O10	C[N@@+]([C@H](CC1)CC2OC(CCC(OC(C[C@@H]3CC4)C[C@@H]4[N@@+]3(C)CC(C=C5)=CC(OC)=C5OC(C)=O)=O)=O)([C@@H]1C2)CC(C=C6)=CC(OC)=C6OC(C)=O	WO9921854	722	+2	-4.068

**Gyermek1999-11**	C40H58N2O10	C[N@+]([C@@H](CC1)CC2OC(CCC(OC(C[C@H]3CC4)C[C@H]4[N@+]3(C)CC(C=C5OC)=CC(OC)=C5OC)=O)=O)([C@H]1C2)CC(C=C6OC)=CC(OC)=C6OC	WO9921854	726	+2	-3.644
**Gyermek1999-12**	C42H62N2O8	C[N@+]([C@@H](CC1)CC2OC(CCC(OC(C[C@H]3CC4)C[C@H]4[N@+]3(C)CC(C=C5)=CC(OCC)=C5OCC)=O)=O)([C@H]1C2)CC(C=C6)=CC(OCC)=C6OCC	WO9921854	722	+2	-0.812
**Gyermek1999-13**	C40H50N2O8	O=C(C1=CC(C(OC(C2)C(CC3)CC[N+]23CC(C=C4OC)=C(C=C4)OC)=O)=CC=C1)OC5C[N+]6(CCC5CC6)CC(C=C7OC)=C(C=C7)OC	WO9921854	686	+2	1.569
**Gyermek1999-14**	C57H86N2O12	O=C(OC(CC1C)CC([N+](C)1CCC(C=C2)=CC(OC(CC)=O)=C2OC(CC)=O)C)CCCC3(CCCC3)CCCC(OC(CC4C)CC([N+](C)4CCC(C=C5)=CC(OC(CC)=O)=C5OC(CC)=O)C)=O	WO9921854	990	+2	0.757
**Gyermek1999-15**	C50H70N2O12	C[N@@+]([C@H](CC1)CC2OC(CCC(OC(C[C@H]3CC4)C[C@H]4[N@+]3(C)CCCC(C=C5)=CC(OC(CC)=O)=C5OC(CC)=O)=O)=O)([C@@H]1C2)CCCC(C=C6)=CC(OC(CC)=O)=C6OC(CC)=O	WO9921854	890	+2	-3.376
**Gyermek1999-16**	C50H58N2O12	C[N@@+]([C@H](CC1)CC2OC(CCC(OC(C[C@H]3CC4)C[C@H]4[N@+]3(C)CC5=CC(C=CC=C6OC(C)=O)=C6C=C5OC(C)=O)=O)=O)([C@@H]1C2)CC7=CC(C=CC=C8OC(C)=O)=C8C=C7OC(C)=O	WO9921854	878	+2	-2.254
**Gyermek1999-17**	C46H58N2O16	C[N@+]([C@@H](CC1)CC2OC(CCC(OC(C[C@@H]3CC4)C[C@@H]4[N@@+]3(C)CC(C=C5OC(C)=O)=CC(OC(C)=O)=C5OC(C)=O)=O)=O)([C@H]1C2)CC(C=C6OC(C)=O)=CC(OC(C)=O)=C6OC(C)=O	WO9921854	894	+2	-7.064
**Gyermek1999-18**	C44H60N2O12	C[N@@+]([C@@](CC1)([H])C[C@@H]2OC(/C=C/C(O[C@@H](C[C@@]3([H])CC4)C[C@@]4([H])[N@+]3(C)CC(C=C5OC)=CC(OC)=C5OC(CC)=O)=O)=O)([C@]1([H])C2)CC(C=C6OC)=CC(OC)=C6OC(CC)=O	WO9921854	808	+2	-3.366
**Gyermek1999-19**	C48H64N2O12	C[N@@+]([C@@](CC1)([H])C[C@@H]2OC(C(CC3)C3C(O[C@@H](C[C@@]4([H])CC5)C[C@@]5([H])[N@+]4(C)CC(C=C6)=CC(OC(CC)=O)=C6OC(CC)=O)=O)=O)([C@]1([H])C2)CC(C=C7)=CC(OC(CC)=O)=C7OC(CC)=O	WO9921854	860	+2	-2.808
**Gyermek1999-20**	C48H66N2O12	C[N@@+]([C@@](CC1)([H])C[C@@H]2OC(CCCCC(O[C@@H](C[C@@]3([H])CC4)C[C@@]4([H])[N@+]3(C)CC(C=C5)=CC(OC(CC)=O)=C5OC(CC)=O)=O)=O)([C@]1([H])C2)CC(C=C6)=CC(OC(CC)=O)=C6OC(CC)=O	WO9921854	862	+2	-2.788
**Gyermek1999-21**	C47H64N2O12	C[N@@+]([C@@](CC1)([H])C[C@@H]2OC(CCCC(O[C@@H](C[C@@]3([H])CC4)C[C@@]4([H])[N@+]3(C)CC(C=C5)=CC(OC(CC)=O)=C5OC(CC)=O)=O)=O)([C@]1([H])C2)CC(C=C6)=CC(OC(CC)=O)=C6OC(CC)=O	WO9921854	848	+2	-2.737
**Gyermek1999-22**	C47H58N2O12	C[N@@+]([C@@](CC1)([H])C[C@H]2OC([C@@H]([C@@]3([H])C[C@]4([H])C=C3)[C@H]4C(O[C@H](C[C@@]5([H])CC6)C[C@@]6([H])[N@+]5(C)CC(C=C7)=CC(OC(C)=O)=C7OC(C)=O)=O)=O)([C@]1([H])C2)CC(C=C8)=CC(OC(C)=O)=C8OC(C)=O	WO9921854	842	+2	-4.015
**Gyermek1999-23**	C43H56N2O12	C[N@@+]([C@@](CC1)([H])C[C@@H]2OC(CCCC(O[C@@H](C[	WO9921854	792	+2	-4.853

		C@@]3([H])CC4)C[C@@]4([H])[N@+]3(C)CC(C=C5)=CC(OC(C)=O)=C5OC(C)=O)=O)=O)([C@]1([H])C2)CC(C=C6)=CC(OC(C)=O)=C6OC(C)=O				
**Gyermek1999-24**	C46H60N2O12	C[N@@+]([C@@](CC1)([H])C[C@@H]2OC(C(CCC3)CC3C(O[C@@H](C[C@@]4([H])CC5)C[C@@]5([H])[N@+]4(C)CC(C=C6)=CC(OC(C)=O)=C6OC(C)=O)=O)=O)([C@]1([H])C2)CC(C=C7)=CC(OC(C)=O)=C7OC(C)=O	WO9921854	832	+2	-3.980
**Gyermek1999-25**	C44H58N2O12	C[N@@+]([C@@](CC1)([H])C[C@@H]2OC(C(CC3)C3C(O[C@@H](C[C@]4([H])[N@+](C)(C([H])CC4)CC(C=C5)=CC(OC(C)=O)=C5OC(C)=O)C)=O)=O)([C@]1([H])C2)CC(C=C6)=CC(OC(C)=O)=C6OC(C)=O	WO9921854	806	+2	-3.17
**Gyermek1999-26**	C47H58N2O12	C[N@@+]([C@@](CC1)([H])C[C@@H]2OC([C@@H]([C@@]3([H])C[C@]4([H])C=C3)[C@H]4C(O[C@@H](C[C@@]5([H])CC6)C[C@@]6([H])[N@+]5(C)CC(C=C7)=CC(OC(C)=O)=C7OC(C)=O)=O)=O)([C@]1([H])C2)CC(C=C8)=CC(OC(C)=O)=C8OC(C)=O	WO9921854	842	+2	-4.015
**Gyermek1999-27**	C44H60N2O10	C[N@@+]([C@@](CC1)([H])C[C@@H]2OC(C(CCC3)CC3C(O[C@@H](C[C@@]4([H])CC5)C[C@@]5([H])[N@+]4(C)CC(C=C6)=CC(OC)=C6OC(C)=O)=O)=O)([C@]1([H])C2)CC(C=C7)=CC(OC)=C7OC(C)=O	WO9921854	776	+2	-2.84
**Gyermek1999-28**	C42H56N2O10	C[N@@+]([C@@](CC1)([H])C[C@@H]2OC(C(CC3)C3C(O[C@@H](C[C@@]4([H])CC5)C[C@@]5([H])[N@+]4(C)CC(C=C6)=CC(OC)=C6OC(C)=O)=O)=O)([C@]1([H])C2)CC(C=C7)=CC(OC)=C7OC(C)=O	WO9921854	748	+2	-3.784
**Gyermek1999-29**	C45H58N2O10	O=C([C@@H]([C@@]1([H])C[C@]2([H])C=C1)[C@H]2C(O[C@@H](C[C@@]3([H])CC4)C[C@@]4([H])[N@+]3(C)CC(C=C5)=CC(OC)=C5OC(C)=O)=O)O[C@H](C[C@]6([H])CC7)C[C@]7([H])[N@@+]6(C)CC(C=C8)=CC(OC)=C8OC(C)=O	WO9921854	786	+2	-2.875
**Gyermek1999-30**	C48H68N2O12	C[N@@+]([C@@](CC1)([H])C[C@@H]2OC(C(CCC3)CC3C(O[C@@H](C[C@@]4([H])CC5)C[C@@]5([H])[N@+]4(C)CC(C=C6OC)=CC(OC)=C6OC(CC)=O)=O)=O)([C@]1([H])C2)CC(C=C7OC)=CC(OC)=C7OC(CC)=O	WO9921854	864	+2	-2.498
**Gyermek1999-31**	C44H60N2O12	C[N@@+]([C@@](CC1)([H])C[C@@H]2OC(C(CC3)C3C(O[C@@H](C[C@@]4([H])CC5)C[C@@]5([H])[N@+]4(C)CC(C=C6OC)=CC(OC)=C6OC(C)=O)=O)=O)([C@]1([H])C2)CC(C=C7OC)=CC(OC)=C7OC(C)=O	WO9921854	808	+2	-4.50
**Gyermek1999-32**	C47H62N2O12	O=C([C@@H]([C@@]1([H])C[C@]2([H])C=C1)[C@H]2C(O[C@@H](C[C@@]3([H])CC4)C[C@@]4([H])[N@+]3(C)CC(C=C5OC)=CC(OC)=C5OC(C)=O)=O)O[C@H](C[C@]6([H])CC7)C[C@]7([H])[N@@+]6(C)CC(C=C8OC)=CC(OC)=C8OC(C)=O	WO9921854	846	+2	-3.591
**Gyermek1999-33**	C47H68N2O12	C[N@@+]([C@@](CC1)([H])C[C@@H]2OC(CCCCCCCC(O[C@@H](C[C@@]3([H])CC4)C[C@@]4([H])[N@+]3(C)CC(C=C5OC)=CC(OC)=C5OC(C)=O)=O)=O)([C@]1([H])C2)CC(C=C6OC)=CC(O	WO9921854	852	+2	-2.893

		C)=C6OC(C)=O				
**Gyermek1999-34**	C45H68N2O10	C[N@@+]([C@@](CC1)([H])C[C@@H]2OC(CCCCCCCC(O[C@@H](C[C@@]3([H])CC4)C[C@@]4([H])[N@+]3(C)CC(C=C5OC)=CC(OC)=C5OC)=O)=O)([C@]1([H])C2)CC(C=C6OC)=CC(OC)=C6OC	WO9921854	796	+2	-1.753
**Gyermek1999-35**	C43H58N2O8	C[N@@+]([C@@](CC1)([H])C[C@@H]2OC([C@@H]([C@@]3([H])C[C@]4([H])C=C3)[C@H]4C(O[C@@H](C[C@@]5([H])CC6)C[C@@]6([H])[N@+]5(C)CC(C=C7OC)=C(C=C7)OC)=O)=O)([C@]1([H])C2)CC(C=C8OC)=C(C=C8)OC	WO9921854	730	+2	-1.035
**Gyermek1999-36**	C50H68N2O12	C[N@@+]([C@H](CCC1)CC2OC(C(CC3)C3C(OC(C[C@H]4CCC5)C[C@H]5[N@+]4(C)CC(C=C6)=CC(OC(CC)=O)=C6OC(CC)=O)=O)=O)([C@@H]1C2)CC(C=C7)=CC(OC(CC)=O)=C7OC(CC)=O	WO9921854	888	+2	-1.69
**Gyermek1999-37**	C51H72N2O12	C[N@@+]([C@H](CCC1)CC2OC(CCCCCC(OC(C[C@H]3CCC4)C[C@H]4[N@+]3(C)CC(C=C5)=CC(OC(CC)=O)=C5OC(CC)=O)=O)=O)([C@@H]1C2)CC(C=C6)=CC(OC(CC)=O)=C6OC(CC)=O	WO9921854	904	+2	-1.141
**Gyermek1999-38**	C50H70N2O12	C[N@+]([C@@H](CCC1)CC2OC(CCCCC(OC(C[C@@H]3CCC4)C[C@@H]4[N@@+]3(C)CC(C=C5)=CC(OC(CC)=O)=C5OC(CC)=O)=O)=O)([C@H]1C2)CC(C=C6)=CC(OC(CC)=O)=C6OC(CC)=O	WO9921854	890	+2	-1.67
**Gyermek1999-39**	C49H68N2O12	C[N@@+]([C@H](CCC1)CC2OC(CCCC(OC(C[C@H]3CCC4)C[C@H]4[N@+]3(C)CC(C=C5)=CC(OC(CC)=O)=C5OC(CC)=O)=O)=O)([C@@H]1C2)CC(C=C6)=CC(OC(CC)=O)=C6OC(CC)=O	WO9921854	876	+2	-1.619
**Gyermek1999-40**	C46H60N2O12	C[N@+]([C@@H](CCC1)CC2OC(C(CC3)C3C(OC(C[C@@H]4CCC5)C[C@@H]5[N@@+]4(C)CC(C=C6)=CC(OC(C)=O)=C6OC(C)=O)=O)=O)([C@H]1C2)CC(C=C7)=CC(OC(C)=O)=C7OC(C)=O	WO9921854	832	+2	-3.806
**Gyermek1999-41**	C48H66N2O12	C[N@+]([C@@H](CCC1)CC2OC(CCCCCCC(OC(C[C@@H]3CCC4)C[C@@H]4[N@@+]3(C)CC(C=C5)=CC(OC(C)=O)=C5OC(C)=O)=O)=O)([C@H]1C2)CC(C=C6)=CC(OC(C)=O)=C6OC(C)=O	WO9921854	862	+2	-2.728
**Gyermek1999-42**	C47H64N2O12	C[N@@+]([C@H](CCC1)CC2OC(CCCCCC(OC(C[C@H]3CCC4)C[C@H]4[N@+]3(C)CC(C=C5)=CC(OC(C)=O)=C5OC(C)=O)=O)=O)([C@@H]1C2)CC(C=C6)=CC(OC(C)=O)=C6OC(C)=O	WO9921854	848	+2	-3.257
**Gyermek1999-43**	C46H62N2O12	C[N@+]([C@@H](CCC1)CC2OC(CCCCC(OC(C[C@@H]3CCC4)C[C@@H]4[N@@+]3(C)CC(C=C5)=CC(OC(C)=O)=C5OC(C)=O)=O)=O)([C@H]1C2)CC(C=C6)=CC(OC(C)=O)=C6OC(C)=O	WO9921854	834	+2	-3.786
**Gyermek1999-44**	C45H60N2O12	C[N@@+]([C@H](CCC1)CC2OC(CCCC(OC(C[C@H]3CCC4)C[C@H]4[N@+]3(C)CC(C=C5)=CC(OC(C)=O)=C5OC(C)=O)=O)=O)([C@@H]1C2)CC(C=C6)=CC(OC(C)=O)=C6OC(C)=O	WO9921854	820	+2	-3.735
**Gyermek1999-45**	C43H54N2O10	O=C([C@@H]([C@]1([H])C[C@@]2([H])C=C1)[C@H]2C(OC(C3)C(CC4)CC[N+]34CC(C=C5)=CC(OC)=C5OC(C)=O)=O)OC(C6)C(CC7)CC[N+]67CC(C=C8)=CC(OC)=C8OC(C)=O	WO9921854	758	+2	-0.325
**Gyermek1999-46**	C38H54N2O8	C[N@@+]([C@H](CC1)CC2OC(CCC(OC(C[C@H]3CC4)C[C@H]4[N@+]3(C)CC(C=C5OC)=C(C=C5)OC)=O)=O)([C@@H]1C2)CC(C=C6OC)=C(C=C6)OC	WO9921854	666	+2	-2.228

**Gyermek1999-47**	C40H58N2O10	C[N@+]([C@@H](CC1)CC2OC(CCC(OC(C[C@H]3CC4)C[C@H]4[N@+]3(C)CC(C=C5OC)=CC(OC)=C5OC)=O)=O)([C@H]1C2)CC(C=C6OC)=CC(OC)=C6OC	WO9921854	726	+2	-3.644
**Gyermek1999-48**	C42H54N2O12	C[N@@+]([C@@](CC1)([H])C[C@@H]2OC(CCC(O[C@H](C[C@]3([H])CC4)C[C@]4([H])[N@@+]3(C)CC(C=C5)=CC(OC(C)=O)=C5OC(C)=O)=O)=O)([C@]1([H])C2)CC(C=C6)=CC(OC(C)=O)=C6OC(C)=O	WO9921854	778	+2	-5.208
**Gyermek2002-1**	C41H60N2O6	C[N+]1(C)C2CCC1CC(OC(C(C3=CC=CC=C3)COCCCCCOCC(C(OC4CC5CCC(C4)[N+]5(C)C)=O)C6=CC=CC=C6)=O)C2	10.1016/s0163-7258(02)00296-6	676	+2	-3.911
**Gyermek2002-2**	C39H56N2O6	C[N@+]1(CCCCC[N@@+]2(C)C3CCC2C[C@@H](OC(C(C4=CC=CC=C4)CO)=O)C3)C5CCC1C[C@H](OC(C(C6=CC=CC=C6)CO)=O)C5	10.1016/s0163-7258(02)00296-6	648	+2	-5.156
**Gyermek2002-3**	C44H66N2O6	C[N@+]1(CCCCCCCCCC[N@@+]2(C)C3CCC2C[C@@H](OC(C(C4=CC=CC=C4)CO)=O)C3)C5CCC1C[C@H](OC(C(C6=CC=CC=C6)CO)=O)C5	10.1016/s0163-7258(02)00296-6	718	+2	-2.512
**Gyermek2002-4**	C38H46N2O4	C[N@+]1(CC2=CC=C(C[N@@+]3(C)C4CCC3C[C@@H](OC(C5=CC=CC=C5)=O)C4)C=C2)C6CCC1C[C@H](OC(C7=CC=CC=C7)=O)C6	10.1016/s0163-7258(02)00296-6	594	+2	-0.588
**Gyermek2002-5**	C40H50N2O6	C[N@+]1(CC2=CC=C(C[N@@+]3(C)C4CCC3C[C@@H](OC(C(C5=CC=CC=C5)O)=O)C4)C=C2)C6CCC1C[C@H](OC(C(C7=CC=CC=C7)O)=O)C6	10.1016/s0163-7258(02)00296-6	654	+2	-2.699
**Gyermek2002-6**	C42H54N2O6	C[N@+]1(CC2=CC=C(C[N@@+]3(C)C4CCC3C[C@@H](OC(C(C5=CC=CC=C5)CO)=O)C4)C=C2)C6CCC1C[C@H](OC(C(C7=CC=CC=C7)CO)=O)C6	10.1016/s0163-7258(02)00296-6	682	+2	-2.956
**Gyermek2002-7**	C22H36N2O4	C[N+]1(C)C2CCC1CC(OC(/C=C\C(OC3CC4CCC(C3)[N+]4(C)C)=O)=O)C2	10.1016/s0163-7258(02)00296-6	392	+2	-7.12
**Gyermek2002-8**	C26H38N2O4	C[N+]1(C)C2CCC1CC(OC(C3=CC=C(C(OC4CC5CCC(C4)[N+]5(C)C)=O)C=C3)=O)C2	10.1016/s0163-7258(02)00296-6	442	+2	-5.824
**Gyermek2002-9**	C34H44N2O4	C[N@@+]1(CC2=CC=CC=C2)C3CCC1C[C@H](OC(/C=C\C(O[C@H]4CC5CCC(C4)[N@+]5(C)CC6=CC=CC=C6)=O)=O)C3	10.1016/s0163-7258(02)00296-6	544	+2	-1.884
**Gyermek2002-10**	C38H44Br2N2O4	C[N@@+]1(CC2=CC(Br)=CC=C2)C3CCC1C[C@H](OC(C4=CC=C(C(O[C@H]5CC6CCC(C5)[N@+]6(C)CC7=CC=CC(Br)=C7)=O)C=C4)=O)C3	10.1016/s0163-7258(02)00296-6	750	+2	1.138
**Gyermek2002-11**	C38H52N2O4	C[N@@+]1(CC)C2CCC1C[C@H](OC([C@@H]3C(C=CC=C4)=C4[C@](CC3)(C5=CC=CC=C5)C(O[C@H]6CC7CCC(C6)[N@+]7(CC)C)=O)=O)C2	10.1016/s0163-7258(02)00296-6	600	+2	-3.416
**Gyermek2002-12**	C22H38N2O4	C[N+]1(C)C2CCC1CC(OC(CCC(OC3CC4CCC(C3)[N+]4(C)C)=O)=O)C2	10.1016/s0163-7258(02)00296-6	394	+2	-7.84
**Gyermek2002-13**	C23H40N2O4	C[N+]1(C)C2CCC1CC(OC(CCCC(OC3CC4CCC(C3)[N+]4(C)C)=O)=O)C2	10.1016/s0163-7258(02)00296-6	408	+2	-7.125
**Gyermek2002-14**	C24H42N2O4	C[N+]1(C)C2CCC1CC(OC(CCCCC(OC3CC4CCC(C3)[N+]4(C)C)=O)=O)C2	10.1016/s0163-7258(02)00296-6	422	+2	-7.176

**Gyermek2002-15**	C25H44N2O4	C[N+]1(C)C2CCC1CC(OC(CCCCCC(OC3CC4CCC(C3)[N+]4(C)C)=O)=O)C2	10.1016/s0163-7258(02)00296-6	436	+2	-6.647
**Gyermek2002-16**	C26H46N2O4	C[N+]1(C)C2CCC1CC(OC(CCCCCCC(OC3CC4CCC(C3)[N+]4(C)C)=O)=O)C2	10.1016/s0163-7258(02)00296-6	450	+2	-6.118
**Gyermek2002-17**	C27H48N2O4	C[N+]1(C)C2CCC1CC(OC(CCCCCCCC(OC3CC4CCC(C3)[N+]4(C)C)=O)=O)C2	10.1016/s0163-7258(02)00296-6	464	+2	-5.589
**Gyermek2002-18**	C28H50N2O4	C[N+]1(C)C2CCC1CC(OC(CCCCCCCCC(OC3CC4CCC(C3)[N+]4(C)C)=O)=O)C2	10.1016/s0163-7258(02)00296-6	478	+2	-5.06
**Gyermek2002-19**	C29H52N2O4	C[N+]1(C)C2CCC1CC(OC(CCCCCCCCCC(OC3CC4CCC(C3)[N+]4(C)C)=O)=O)C2	10.1016/s0163-7258(02)00296-6	492	+2	-4.531
**Gyermek2002-20**	C30H54N2O4	C[N+]1(C)C2CCC1CC(OC(CCCCCCCCCCC(OC3CC4CCC(C3)[N+]4(C)C)=O)=O)C2	10.1016/s0163-7258(02)00296-6	506	+2	-4.002
**Gyermek2002-21**	C31H56N2O4	C[N+]1(C)C2CCC1CC(OC(CCCCCCCCCCCC(OC3CC4CCC(C3)[N+]4(C)C)=O)=O)C2	10.1016/s0163-7258(02)00296-6	520	+2	-2.473
**Gyermek2002-22**	C32H58N2O4	C[N+]1(C)C2CCC1CC(OC(CCCCCCCCCCCCC(OC3CC4CCC(C3)[N+]4(C)C)=O)=O)C2	10.1016/s0163-7258(02)00296-6	534	+2	-2.944
**Gyermek2002-23**	C35H44Cl4N2O4	C[N@@+]1(CC2=C(Cl)C=CC=C2Cl)C3CCC1C[C@H](OC(CCCC(O[C@H]4CC5CCC(C4)[N@+]5(C)CC6=C(Cl)C=CC=C6Cl)=O)=O)C3	10.1016/s0163-7258(02)00296-6	696	+2	0.963
**Gyermek2002-24**	C22H36NO8	O=C(CCCCC(O)=O)OCC[N@+]([C@H](CC1)CC2OC(CCCCC(O)=O)=O)([C@@H]1C2)C	10.1016/s0163-7258(02)00296-6	442	+2	-1.907
**Gyermek2002-25**	C22H42N2O4	O=C(CCCCC(OCC[N+]1(CCCCC1)C)=O)OCC[N+]2(CCCCC2)C	10.1016/s0163-7258(02)00296-6	398	+2	-2.475
**Gyermek2002-26**	C34H50N2O4	O=C(CCCCC(OCC[N+]1(CCCCC1)CC2=CC=CC=C2)=O)OCC[N+]3(CCCCC3)CC4=CC=CC=C4	10.1016/s0163-7258(02)00296-6	550	+2	2.278
**Gyermek2002-27**	C28H46N2O8	CC(OCC[N@+]([C@H](CC1)CC2OC(CCC(OC(C[C@H]3CC4)C[C@H]4[N@+]3(C)CCOC(C)=O)=O)=O)([C@@H]1C2)C)=O	10.1016/s0163-7258(02)00296-6	538	+2	-4.433
**Gyermek2002-28**	C38H50N2O8	O=C(C(OCC[N@@+]([C@@H](CC1)CC2OC(CC3=CC=CC=C3)=O)([C@H]1C2)C)=O)OCC[N@+]([C@H](CC4)CC5OC(CC6=CC=CC=C6)=O)([C@@H]4C5)C	10.1016/s0163-7258(02)00296-6	662	+2	-1.588
**Gyermek2002-29**	C44H62N2O8	O=C(CCCCCCC(OCC[N@+]([C@H](CC1)CC2OC(CC3=CC=CC=C3)=O)([C@@H]1C2)C)=O)OCC[N@@+]([C@@H](CC4)CC5OC(CC6=CC=CC=C6)=O)([C@H]4C5)C	10.1016/s0163-7258(02)00296-6	746	+2	0.205
**Gyermek2002-30**	C44H62N2O8	O=C(CCCCC(OCCC[N@+]([C@H](CC1)CC2OC(CC3=CC=CC=C3)=O)([C@@H]1C2)C)=O)OCCC[N@@+]([C@@H](CC4)CC5OC(CC6=CC=CC=C6)=O)([C@H]4C5)C	10.1016/s0163-7258(02)00296-6	746	+2	-1.211
**Gyermek2002-31**	C44H62N2O8	O=C(CCCCC(OCC[N@+]([C@H](CC1)CC2OC(CCC3=CC=CC=C3)=O)([C@@H]1C2)C)=O)OCC[N@@+]([C@@H](CC4)CC5OC(CCC6=CC=CC=C6)=O)([C@H]4C5)C	10.1016/s0163-7258(02)00296-6	746	+2	0.065
**Gyermek2002-32**	C41H56N2O8	O=C(CCCCCC(OCC[N@+]([C@H](CC1)CC2OC(C3=CC=CC=C3)=O)([C@@H]1C2)C)=O)OCC[N@@+]([C@@H](CC4)CC5OC(C6=CC=CC=C6)=O)([C@H]4C5)C	10.1016/s0163-7258(02)00296-6	704	+2	0.258

**Gyermek2002-33**	C32H54N2O8	O=C(CCCCCCC(OCC[N@+]([C@H](CC1)CC2OC(C)=O)([C@@H]1C2)C)=O)OCC[N@@+]([C@@H](CC3)CC4OC(C)=O)([C@H]3C4)C	10.1016/s0163-7258(02)00296-6	594	+2	-3.071
**Gyermek2002-34**	C42H58N2O8	O=C(CCC(OCCC[N@+]([C@H](CC1)CC2OC(CC3=CC=CC=C3)=O)([C@@H]1C2)C)=O)OCCC[N@+]4([C@@H](CC[C@@H]4CC5)[C@@H]5OC(CC6=CC=CC=C6)=O)C	10.1016/s0163-7258(02)00296-6	718	+2	0.504
**Gyermek2002-35**	C42H58N2O8	O=C(C(OCCCC[N@+]([C@H](CC1)CC2OC(CC3=CC=CC=C3)=O)([C@@H]1C2)C)=O)OCCCC[N@@+]([C@@H](CC4)CC5OC(CC6=CC=CC=C6)=O)([C@H]4C5)C	10.1016/s0163-7258(02)00296-6	718	+2	-3.704
**Gyermek2002-36**	C30H50N2O8	O=C(CCCCC(OCC[N@+]([C@H](CC1)CC2OC(C)=O)([C@@H]1C2)C)=O)OCC[N@@+]([C@@H](CC3)CC4OC(C)=O)([C@H]3C4)C	10.1016/s0163-7258(02)00296-6	566	+2	-4.129
**Gyermek2002-37**	C43H60N2O8	O=C(CCCC(OCCC[N@+]([C@H](CC1)CC2OC(CC3=CC=CC=C3)=O)([C@@H]1C2)C)=O)OCCC[N@@+]([C@@H](CC4)CC5OC(CC6=CC=CC=C6)=O)([C@H]4C5)C	10.1016/s0163-7258(02)00296-6	732	+2	-1.16
**Gyermek2002-38**	C37H48N2O8	O=C(C1=CC=CC=C1)OCC[N@@+]([C@@H](CC2)CC3OC(CC(OC(C[C@H]4CC5)C[C@H]5[N@+]4(C)CCOC(C6=CC=CC=C6)=O)=O)=O)([C@H]2C3)C	10.1016/s0163-7258(02)00296-6	648	+2	-1.339
**Gyermek2002-39**	C46H66N2O8	O=C(CCCCCCC(OCCC[N@@+]([C@@H](CC1)CC2OC(CC3=CC=CC=C3)=O)([C@H]1C2)C)=O)OCCC[N@+]([C@H](CC4)CC5OC(CC6=CC=CC=C6)=O)([C@@H]4C5)C	10.1016/s0163-7258(02)00296-6	774	+2	-0.153
**Gyermek2002-40**	C40H54N2O8	O=C(CCCCC(OCC[N@+]([C@H](CC1)CC2OC(C3=CC=CC=C3)=O)([C@@H]1C2)C)=O)OCC[N@@+]([C@@H](CC4)CC5OC(C6=CC=CC=C6)=O)([C@H]4C5)C	10.1016/s0163-7258(02)00296-6	690	+2	-0.271
**Gyermek2002-41**	C41H56N2O8	O=C(CC(OCCC[N@+]([C@H](CC1)CC2OC(CC3=CC=CC=C3)=O)([C@@H]1C2)C)=O)OCCC[N@@+]([C@@H](CC4)CC5OC(CC6=CC=CC=C6)=O)([C@H]4C5)C	10.1016/s0163-7258(02)00296-6	704	+2	-1.87
**Gyermek2002-42**	C41H56N2O8	O=C(CCCC(OCC[N@+]([C@@](CC1)([H])C[C@@H]2OC(CC3=CC=CC=C3)=O)([C@]1([H])C2)C)=O)OCC[N@@+]([C@@](CC4)([H])C[C@@H]5OC(CC6=CC=CC=C6)=O)([C@]4([H])C5)C	10.1016/s0163-7258(02)00296-6	704	+2	-0.802
**Gyermek2002-43**	C39H52N2O8	O=C(CC(OCC[N@@+]([C@@H](CC1)CC2OC(CC3=CC=CC=C3)=O)([C@H]1C2)C)=O)OCC[N@+]([C@H](CC4)CC5OC(CC6=CC=CC=C6)=O)([C@@H]4C5)C	10.1016/s0163-7258(02)00296-6	676	+2	-1.512
**Gyermek2002-44**	C43H60N2O8	O=C(CCCCCC(OCC[N@+]([C@H](CC1)CC2OC(CC3=CC=CC=C3)=O)([C@@H]1C2)C)=O)OCC[N@@+]([C@@H](CC4)CC5OC(CC6=CC=CC=C6)=O)([C@H]4C5)C	10.1016/s0163-7258(02)00296-6	732	+2	-0.324
**Gyermek2002-45**	C38H50N2O8	O=C(CCC(OCC[N@+]([C@H](CC1)CC2OC(C3=CC=CC=C3)=O)([C@@H]1C2)C)=O)OCC[N@@+]([C@@H](CC4)CC5OC(C6=CC=CC=C6)=O)([C@H]4C5)C	10.1016/s0163-7258(02)00296-6	662	+2	-0.575
**Gyermek2002-46**	C42H58N2O8	O=C(CCCCC(OCC[N@+]([C@H](CC1)CC2OC(CC3=CC=CC=C3)=O)([C@@H]1C2)C)=O)OCC[N@@+]([C@@H](CC4)CC5OC(CC6=CC=CC=C6)=O)([C@H]4C5)C	10.1016/s0163-7258(02)00296-6	718	+2	-0.853
**Gyermek2002-47**	C40H54N2O8	O=C(CCC(OCC[N@+]([C@H](CC1)CC2OC(CC3=CC=CC=C3)=O)	10.1016/s0163-	690	+2	-1.157

		([C@@H]1C2)C)=O)OCC[N@@+]([C@@H](CC4)CC5OC(CC6=C C=CC=C6)=O)([C@H]4C5)C	7258(02)00296-6			
**Gyermek2002-48**	C42H58N2O8	O=C(CCCCCCC(OCC[N@+]([C@H](CC1)CC2OC(C3=CC=CC=C3)=O)([C@@H]1C2)C)=O)OCC[N@@+]([C@@H](CC4)CC5OC(C6 =CC=CC=C6)=O)([C@H]4C5)C	10.1016/s0163-7258(02)00296-6	718	+2	0.787
**Gyermek2002-49**	C22H42N2O4	O=C(CCCCC(OCC[N+]1(CCCCC1)C)=O)OCC[N+]2(CCCCC2)C	10.1016/s0163-7258(02)00296-6	398	+2	-2.475
**Gyermek2002-50**	C42H62N2O4	C[N@+]([C@@H](CC1)CC2OC(CC3=CC=CC=C3)=O)([C@H]1C2)CCCCCCCCCC[N@+]([C@H](CC4)CC5OC(CC6=CC=CC=C6)=O)([C@@H]4C5)C	10.1016/s0163-7258(02)00296-6	658	+2	-0.726
**Gyermek2002-51**	C28H54N2O2	C[N@+]([C@@](CC1)([H])C[C@@H]2O)([C@]1([H])C2)CCCCC CCCCCCC[N@+]([C@](CC3)([H])C[C@H]4O)([C@@]3([H])C4)C	10.1016/s0163-7258(02)00296-6	450	+2	-4.836
**Gyermek2002-52**	C25H48N2O2	C[N@+]([C@@](CC1)([H])C[C@@H]2O)([C@]1([H])C2)CCCCC CCCC[N@+]([C@](CC3)([H])C[C@H]4O)([C@@]3([H])C4)C	10.1016/s0163-7258(02)00296-6	408	+2	-6.423
**Gyermek2002-53**	C24H46N2O2	C[N@+]([C@@](CC1)([H])C[C@@H]2O)([C@]1([H])C2)CCCCC CCC[N@+]([C@](CC3)([H])C[C@H]4O)([C@@]3([H])C4)C	10.1016/s0163-7258(02)00296-6	394	+2	-6.952
**Gyermek2002-54**	C23H44N2O2	C[N@+]([C@@](CC1)([H])C[C@@H]2O)([C@]1([H])C2)CCCCC CC[N@+]([C@](CC3)([H])C[C@H]4O)([C@@]3([H])C4)C	10.1016/s0163-7258(02)00296-6	380	+2	-7.481
**Gyermek2002-55**	C28H54N2	C[N@@+]1([C@H]2CC[C@@H]1CCC2)CCCCCCCCCCCC[N@@ +]3([C@@H]4CC[C@H]3CCC4)C	10.1016/s0163-7258(02)00296-6	418	+2	-0.662
**Gyermek2002-56**	C27H52N2	C[N@@+]1([C@H]2CC[C@@H]1CCC2)CCCCCCCCCCC[N@@+]3([C@@H]4CC[C@H]3CCC4)C	10.1016/s0163-7258(02)00296-6	404	+2	-1.191
**Gyermek2002-57**	C26H50N2	C[N@@+]1([C@H]2CC[C@@H]1CCC2)CCCCCCCCCC[N@@+]3 ([C@@H]4CC[C@H]3CCC4)C	10.1016/s0163-7258(02)00296-6	390	+2	-1.72
**Gyermek2002-58**	C25H48N2	C[N@+]1([C@@H]2CC[C@H]1CCC2)CCCCCCCCC[N@+]3([C@ H]4CC[C@@H]3CCC4)C	10.1016/s0163-7258(02)00296-6	376	+2	-2.249
**Gyermek2002-59**	C24H46N2	C[N@@+]1([C@H]2CC[C@@H]1CCC2)CCCCCCCC[N@@+]3([C @@H]4CC[C@H]3CCC4)C	10.1016/s0163-7258(02)00296-6	362	+2	-2.778
**Gyermek2002-60**	C23H44N2	C[N@@+]1([C@H]2CC[C@@H]1CCC2)CCCCCCC[N@@+]3([C @@H]4CC[C@H]3CCC4)C	10.1016/s0163-7258(02)00296-6	348	+2	-3.307
**Gyermek2002-61**	C22H42N2	C[N@@+]1([C@H]2CC[C@@H]1CCC2)CCCCCC[N@@+]3([C@ @H]4CC[C@H]3CCC4)C	10.1016/s0163-7258(02)00296-6	334	+2	-3.836
**Gyermek2002-62**	C21H40N2	C[N@+]1([C@@H]2CC[C@H]1CCC2)CCCCC[N@+]3([C@H]4CC [C@@H]3CCC4)C	10.1016/s0163-7258(02)00296-6	320	+2	-4.365
**Gyermek2002-63**	C19H36N2	C[N@+]1([C@H]2CCC[C@@H]1CC2)CCC[N@+]3([C@@H]4CC C[C@H]3CC4)C	10.1016/s0163-7258(02)00296-6	292	+2	-3.943
**Gyermek2002-64**	C20H38N2	C[N@+]1([C@H]2CCC[C@@H]1CC2)CCCC[N@+]3([C@@H]4C CC[C@H]3CC4)C	10.1016/s0163-7258(02)00296-6	306	+2	-4.894
**Gyermek2002-65**	C22H42N2O2	C[N@+]([C@@](CC1)([H])C[C@H]2O)([C@]1([H])C2)CCCCCC[N@@+]([C@](CC3)([H])C[C@H]4O)([C@@]3([H])C4)C	10.1016/s0163-7258(02)00296-6	366	+2	-8.01
**Gyermek2002-66**	C26H50N2O2	C[N@+]([C@@H](CC1)CC2O)([C@H]1C2)CCCCCCCCCC[N@+] ([C@H](CC3)CC4O)([C@@H]3C4)C	10.1016/s0163-7258(02)00296-6	422	+2	-5.894

**Rocuronium**	C32H53N2O4	O=C(C)O[C@@H]([C@H]1[N+]2(CCCC2)CC=C)[C@](CC3)(C)[C@](C1)([H])[C@@](CC4)([H])[C@@]3([H])[C@@]([C@]4([H])C[C@@H]5O)(C)C[C@@H]5N6CCOCC6	10.2174/1389557054023215	529	+1	2.433
**Vecuronium**	C34H57N2O4	C[C@]([C@H]1OC(C)=O)(CC2)[C@](C[C@@H]1[N+]3(C)CCCCC3)([H])[C@@](CC4)([H])[C@@]2([H])[C@](C[C@@H]5N6CCCCC6)(C)[C@]4([H])C[C@@H]5OC(C)=O	10.2174/1389557054023215	557	+1	4.334
**Pipecuronium**	C35H62N4O4	C[C@]([C@H]1OC(C)=O)(CC2)[C@](C[C@@H]1N3CC[N+](C)(C)CC3)([H])[C@@](CC4)([H])[C@@]2([H])[C@](C[C@@H]5N6CC[N+](C)(C)CC6)(C)[C@]4([H])C[C@@H]5OC(C)=O	10.2174/1389557054023215	602	+2	0.628
**Pancuronium**	C35H60N2O4	C[C@]([C@H]1OC(C)=O)(CC2)[C@](C[C@@H]1[N+]3(C)CCCCC3)([H])[C@@](CC4)([H])[C@@]2([H])[C@](C[C@@H]5[N+]6(C)CCCCC6)(C)[C@]4([H])C[C@@H]5OC(C)=O	10.2174/1389557054023215	572	+2	1.206
**Jindal2001-DPJ-489**	C32H54N2O2	C[C@]12C(C[C@@H]([N+]3(C)CCCC3)CC2)=CCC4C1CC[C@@]5(C)C4C[C@H]([N+]6(C)CCCCC6)[C@@H]5OC(C)=O	10.1016/s0223-5234(00)01205-8	498	+2	-0.732
**Jindal2002-DPJ-494**	C30H53N3O	C[C@]12C(C[C@@H]([N+]3(C)CCCC3)CC2)=CCC4C1CC[C@@]5(C)C4C[C@H](N6CC[N+](C)(C)CC6)[C@@H]5O	10.1016/s0223-5234(02)01413-7	471	+2	-1.923
**Jindal2002-DPJ-496**	C32H55N3O2	C[C@]12C(C[C@@H]([N+]3(C)CCCC3)CC2)=CCC4C1CC[C@@]5(C)C4C[C@H](N6CC[N+](C)(C)CC6)[C@@H]5OC(C)=O	10.1016/s0223-5234(02)01413-7	513	+2	-1.021
**Hu2012-8a**	C29H52N2O3	C[C@@]12C(CCC3C2CC[C@@]4(C)C3C[C@H]([N+]5(C)CCOCC5)[C@@H]4OC(C)=O)C[C@@H]([N+](C)(C)C)CC1	10.1016/j.ejmech.2012.07.048	476	+2	-1.166
**Hu2012-8b**	C31H54N2O3	C[C@@]12C(CCC3C2CC[C@@]4(C)C3C[C@H]([N+]5(C)CCOCC5)[C@@H]4OC(C)=O)C[C@@H]([N+]6(C)CCCC6)CC1	10.1016/j.ejmech.2012.07.048	502	+2	0.099
**Hu2012-8c**	C32H57N3O3	C[C@@]12C(CCC3C2CC[C@@]4(C)C3C[C@H]([N+]5(C)CCOCC5)[C@@H]4OC(C)=O)C[C@@H](N6CC[N+](C)(C)CC6)CC1	10.1016/j.ejmech.2012.07.048	531	+2	1.565
**Hu2012-8d**	C32H56N2O3	C[C@@]12C(CCC3C2CC[C@@]4(C)C3C[C@H]([N+]5(C)CCOCC5)[C@@H]4OC(C)=O)C[C@@H]([N+]6(C)CCCCC6)CC1	10.1016/j.ejmech.2012.07.048	516	+2	0.658
**Hu2012-8e**	C31H54N2O4	C[C@@]12C(CCC3C2CC[C@@]4(C)C3C[C@H]([N+]5(C)CCOCC5)[C@@H]4OC(C)=O)C[C@@H]([N+]6(C)CCOCC6)CC1	10.1016/j.ejmech.2012.07.048	518	+2	1.004
**Hu2012-8f**	C34H54N2O3	C[C@@]12C(CCC3C2CC[C@@]4(C)C3C[C@H]([N+]5(C)CCOCC5)[C@@H]4OC(C)=O)C[C@@H]([N+](C)(C6=CC=CC=C6)C)CC1	10.1016/j.ejmech.2012.07.048	538	+2	1.594
**Hu2012-8g**	C35H56N2O3	C[C@@]12C(CCC3C2CC[C@@]4(C)C3C[C@H]([N+]5(C)CCOCC5)[C@@H]4OC(C)=O)C[C@@H]([N+](C)(CC6=CC=CC=C6)C)CC1	10.1016/j.ejmech.2012.07.048	552	+2	1.253
**Hu2015-24a**	C32H56N2O3	C[C@@]12C(CCC3C2CC[C@@]4(C)C3C[C@H]([N+]5(C)CCCCC5)[C@@H]4OC(C)=O)C[C@@H]([N+]6(C)CCOCC6)CC1	10.1016/j.steroids.2015.01.008	516	+2	0.658
**Hu2015-24b**	C31H54N2O3	C[C@@]12C(CCC3C2CC[C@@]4(C)C3C[C@H]([N+]5(C)CCCC5)[C@@H]4OC(C)=O)C[C@@H]([N+]6(C)CCOCC6)CC1	10.1016/j.steroids.2015.01.008	502	+2	0.099
**Hu2015-24c**	C32H57N3O3	C[C@@]12C(CCC3C2CC[C@@]4(C)C3C[C@H](N5CC[N+](C)(C)CC5)[C@@H]4OC(C)=O)C[C@@H]([N+]6(C)CCOCC6)CC1	10.1016/j.steroids.2015.01.008	531	+2	0.368
**Hu2015-25a**	C33H58N2O2	C[C@@]12C(CCC3C2CC[C@@]4(C)C3C[C@H]([N+]5(C)CCCCC5)[C@@H]4OC(C)=O)C[C@@H]([N+]6(C)CCCCC6)CC1	10.1016/j.steroids.2015.01.008	514	+2	0.312

**Hu2015-25b**	C32H56N2O2	C[C@@]12C(CCC3C2CC[C@@]4(C)C3C[C@H]([N+]5(C)CCCC5)[C@@H]4OC(C)=O)C[C@@H]([N+]6(C)CCCCC6)CC1	10.1016/j.steroids.2015.01.008	500	+2	-0.248
**Hu2015-25c**	C33H59N3O2	C[C@@]12C(CCC3C2CC[C@@]4(C)C3C[C@H](N5CC[N+](C)(C)CC5)[C@@H]4OC(C)=O)C[C@@H]([N+]6(C)CCCCC6)CC1	10.1016/j.steroids.2015.01.008	529	+2	0.022
**Hu2015-26a**	C32H56N2O2	C[C@@]12C(CCC3C2CC[C@@]4(C)C3C[C@H]([N+]5(C)CCCCC5)[C@@H]4OC(C)=O)C[C@@H]([N+]6(C)CCCC6)CC1	10.1016/j.steroids.2015.01.008	500	+2	-0.248
**Hu2015-26b**	C31H54N2O2	C[C@@]12C(CCC3C2CC[C@@]4(C)C3C[C@H]([N+]5(C)CCCC5)[C@@H]4OC(C)=O)C[C@@H]([N+]6(C)CCCC6)CC1	10.1016/j.steroids.2015.01.008	486	+2	-0.807
**Hu2015-26c**	C32H57N3O2	C[C@@]12C(CCC3C2CC[C@@]4(C)C3C[C@H](N5CC[N+](C)(C)CC5)[C@@H]4OC(C)=O)C[C@@H]([N+]6(C)CCCC6)CC1	10.1016/j.steroids.2015.01.008	515	+2	-0.537
**Hu2015-27a**	C33H59N3O2	C[C@@]12C(CCC3C2CC[C@@]4(C)C3C[C@H]([N+]5(C)CCCCC5)[C@@H]4OC(C)=O)C[C@@H](N6CC[N+](C)(C)CC6)CC1	10.1016/j.steroids.2015.01.008	529	+2	1.219
**Hu2015-27b**	C32H57N3O2	C[C@@]12C(CCC3C2CC[C@@]4(C)C3C[C@H]([N+]5(C)CCCC5)[C@@H]4OC(C)=O)C[C@@H](N6CC[N+](C)(C)CC6)CC1	10.1016/j.steroids.2015.01.008	515	+2	0.660
**Hu2015-27c**	C33H60N4O2	C[C@@]12C(CCC3C2CC[C@@]4(C)C3C[C@H](N5CC[N+](C)(C)CC5)[C@@H]4OC(C)=O)C[C@@H](N6CC[N+](C)(C)CC6)CC1	10.1016/j.steroids.2015.01.008	544	+2	0.929
**Hu2015-28**	C31H56N2O	C[C@@]12C(CCC3C2CC[C@@]4(C)C3C[C@H]([N+]5(C)CCCCC5)[C@@H]4O)C[C@@H]([N+]6(C)CCCCC6)CC1	10.1016/j.steroids.2015.01.008	472	+2	-0.591
**Hu2015-29**	C31H57N3O	C[C@@]12C(CCC3C2CC[C@@]4(C)C3C[C@H](N5CC[N+](C)(C)CC5)[C@@H]4O)C[C@@H]([N+]6(C)CCCCC6)CC1	10.1016/j.steroids.2015.01.008	487	+2	-0.880
**Hu2015-30**	C30H49N3O2	C[C@@]12C(CCC3C2CC[C@@]4(C)C3C[C@H](N5C=[N+](C)C=C5)[C@@H]4OC(C)=O)C[C@@H]([N+]6(C)CCCC6)CC1	10.1016/j.steroids.2015.01.008	483	+2	-2.523
**Rao2016-1a**	C37H62N2O2	C[C@@]12C(CCC3C2CC[C@@]4(C)C3C[C@H]([N+]5(CC=C)CCCCC5)[C@@H]4OC(C)=O)C[C@@H]([N+]6(CC=C)CCCCC6)CC1	10.1111/cbdd.12711	566	+2	1.860
**Rao2016-1b**	C37H58N2O2	C[C@@]12C(CCC3C2CC[C@@]4(C)C3C[C@H]([N+]5(CC#C)CCCCC5)[C@@H]4OC(C)=O)C[C@@H]([N+]6(CC#C)CCCCC6)CC1	10.1111/cbdd.12711	562	+2	0.512
**Rao2016-1c**	C34H57N2O2	C[C@@]12C(CCC3C2CC[C@@]4(C)C3C[C@H](N5CCCCC5)[C@@H]4OC(C)=O)C[C@@H]([N+]6(CC=C)CCCCC6)CC1	10.1111/cbdd.12711	525	+1	4.214
**Rao2016-1d**	C34H55N2O2	C[C@@]12C(CCC3C2CC[C@@]4(C)C3C[C@H](N5CCCCC5)[C@@H]4OC(C)=O)C[C@@H]([N+]6(CC#C)CCCCC6)CC1	10.1111/cbdd.12711	523	+1	3.540
**Rao2016-2a**	C37H63N3O2	C[C@@]12C(CCC3C2CC[C@@]4(C)C3C[C@H]([N+]5(CC=C)CCCCC5)[C@@H]4OC(C)=O)C[C@@H](N6CC[N+](C)(CC=C)CC6)CC1	10.1111/cbdd.12711	581	+2	2.767
**Rao2016-2b**	C37H59N3O2	C[C@@]12C(CCC3C2CC[C@@]4(C)C3C[C@H]([N+]5(CC#C)CCCCC5)[C@@H]4OC(C)=O)C[C@@H](N6CC[N+](C)(CC#C)CC6)CC1	10.1111/cbdd.12711	577	+2	1.419
**Rao2016-2c**	C34H58N3O2	C[C@@]12C(CCC3C2CC[C@@]4(C)C3C[C@H](N5CCCCC5)[C@@H]4OC(C)=O)C[C@@H](N6CC[N+](C)(CC=C)CC6)CC1	10.1111/cbdd.12711	540	+1	5.121
**Rao2016-2d**	C34H56N3O2	C[C@@]12C(CCC3C2CC[C@@]4(C)C3C[C@H](N5CCCCC5)[C@@H]4OC(C)=O)C[C@@H](N6CC[N+](C)(CC#C)CC6)CC1	10.1111/cbdd.12711	538	+1	4.447
**Rao2016-2e**	C38H60N3O2	C[C@@]12C(CCC3C2CC[C@@]4(C)C3C[C@H](N5CCCCC5)[C@@H]4OC(C)=O)C[C@@H](N6CC[N+](C)(CC7=CC=CC=C7)CC6)	10.1111/cbdd.12711	590	+1	6.650

		CC1				
**Rao2016-2f**	C39H63N3O2	C[C@@]12C(CCC3C2CC[C@@]4(C)C3C[C@H]([N+]5(C)CCCCC5)[C@@H]4OC(C)=O)C[C@@H](N6CC[N+](C)(CC7=CC=CC=C7)CC6)CC1	10.1111/cbdd.12711	605	+2	3.522
**Rao2016-3a**	C37H63N3O2	C[C@@]12C(CCC3C2CC[C@@]4(C)C3C[C@H](N5CC[N+](C)(CC=C)CC5)[C@@H]4OC(C)=O)C[C@@H]([N+]6(CC=C)CCCCC6)CC1	10.1111/cbdd.12711	581	+2	1.571
**Rao2016-3b**	C37H59N3O2	C[C@@]12C(CCC3C2CC[C@@]4(C)C3C[C@H](N5CC[N+](C)(CC#C)CC5)[C@@H]4OC(C)=O)C[C@@H]([N+]6(CC#C)CCCCC6)CC1	10.1111/cbdd.12711	577	+2	0.222
**Rao2016-3c**	C34H58N3O2	C[C@@]12C(CCC3C2CC[C@@]4(C)C3C[C@H](N5CC[N+](C)(CC=C)CC5)[C@@H]4OC(C)=O)C[C@@H](N6CCCCC6)CC1	10.1111/cbdd.12711	540	+1	5.121
**Rao2016-3d**	C34H56N3O2	C[C@@]12C(CCC3C2CC[C@@]4(C)C3C[C@H](N5CC[N+](C)(CC#C)CC5)[C@@H]4OC(C)=O)C[C@@H](N6CCCCC6)CC1	10.1111/cbdd.12711	538	+1	4.447
**Rao2016-3e**	C38H60N3O2	C[C@@]12C(CCC3C2CC[C@@]4(C)C3C[C@H](N5CC[N+](C)(CC6=CC=CC=C6)CC5)[C@@H]4OC(C)=O)C[C@@H](N7CCCCC7)CC1	10.1111/cbdd.12711	590	+1	6.650
**Rao2016-3f**	C39H63N3O2	C[C@@]12C(CCC3C2CC[C@@]4(C)C3C[C@H](N5CC[N+](C)(CC6=CC=CC=C6)CC5)[C@@H]4OC(C)=O)C[C@@H]([N+]7(C)CCCCC7)CC1	10.1111/cbdd.12711	605	+2	2.326
**Rao2016-3g**	C45H67N3O2	C[C@@]12C(CCC3C2CC[C@@]4(C)C3C[C@H](N5CC[N+](C)(CC6=CC=CC=C6)CC5)[C@@H]4OC(C)=O)C[C@@H]([N+]7(CC8=CC=CC=C8)CCCCC7)CC1	10.1111/cbdd.12711	681	+2	4.944
**Rao2016-4a**	C37H64N4O2	C[C@@]12C(CCC3C2CC[C@@]4(C)C3C[C@H](N5CC[N+](C)(CC=C)CC5)[C@@H]4OC(C)=O)C[C@@H](N6CC[N+](C)(CC=C)CC6)CC1	10.1111/cbdd.12711	596	+2	2.478
**Rao2016-4b**	C37H60N4O2	C[C@@]12C(CCC3C2CC[C@@]4(C)C3C[C@H](N5CC[N+](C)(CC#C)CC5)[C@@H]4OC(C)=O)C[C@@H](N6CC[N+](C)(CC#C)CC6)CC1	10.1111/cbdd.12711	592	+2	1.130
**Rao2016-4g**	C45H68N4O2	C[C@@]12C(CCC3C2CC[C@@]4(C)C3C[C@H](N5CC[N+](C)(CC6=CC=CC=C6)CC5)[C@@H]4OC(C)=O)C[C@@H](N7CC[N+](C)(CC8=CC=CC=C8)CC7)CC1	10.1111/cbdd.12711	696	+2	5.536
**CW 002**	C52H68N2O12	O=C(OCCC[N@+]1(C)CCC(C=C(OC)C(OC)=C2)=C2[C@H]1CC3=CC(OC)=C(OC)C=C3)/C=C/C(OCCC[N@@+]4(C)[C@H](CC5=CC=C(OC)C(OC)=C5)C(C=C(OC)C(OC)=C6)=C6CC4)=O	10.1097/ALN.0b013e3181dc1b5b	912	+2	2.663
**CW 011**	C53H70N2O13	O=C(OCCC[N@+]1(C)CCC(C=C(OC)C(OC)=C2)=C2[C@H]1CC3=CC(OC)=C(OC)C=C3)/C=C\C(OCCC[N@@+]4(C)[C@H](CC5=CC(OC)=C(OC)C(OC)=C5)C(C=C(OC)C(OC)=C6)=C6CC4)=O	10.1097/ALN.0b013e3181dc1b5b	942	+2	2.305
**CW 1759-50**	C43H58N2O10	COC1=C(OC)C=C2C([C@@H](CC3=CC=C(OC)C=C3)[N@+](C)(CCCOC(/C=C\C(OCCC[N+]4(CCOCC4)CC5=CC=C(OC)C(OC)=C5)=O)=O)CC2)=C1	10.1097/ALN.0000000000002408	762	+2	2.268
**Patel1997-1**	C58H80N2O14	O=C(CCCC/C=C/C(OCCC[N@@+]([C@@H](C1=C2)CC(C=C3OC	10.1016/S0928-	1028	+2	3.121

		)=CC(OC)=C3OC)(CCC1=CC(OC)=C2OC)C)=O)OCCC[N@+]([C@@H](C(C4=C5)=CC(OC)=C5OC)CC(C=C6OC)=CC(OC)=C6OC)(CC4)C	0987(97)00020-1			
**Patel1997-4**	C60H88N2O16	COC(C(OC)=CC(CC[N+](CCCOC(CCCC/C=C/C(OCCC[N+](CCC(C=C1OC)=CC(OC)=C1OC)(C)CCC(C=C2OC)=CC(OC)=C2OC)=O)=O)(C)CCC(C=C3OC)=CC(OC)=C3OC)=C4)=C4OC	10.1016/S0928-0987(97)00020-1	1092	+2	-0.256
**Patel1997-5**	C56H82N2O16	COC(C(OC)=CC(CC[N+](CCCOC(CCC(OCCC[N+](CCC(C=C1OC)=CC(OC)=C1OC)(C)CCC(C=C2OC)=CC(OC)=C2OC)=O)=O)(C)CCC(C=C3OC)=CC(OC)=C3OC)=C4)=C4OC	10.1016/S0928-0987(97)00020-1	1038	+2	-1.590
**Patel1997-6**	C60H88N2O14	C[N+](CCCOC(CC/C=C/CCC(OCCC[N+](C)(C)CCC(C=C1OC)=C(C=C1OC)CCC(C=C2OC)=CC(OC)=C2OC)=O)=O)(C)CCC(C=C3OC)=C(C=C3OC)CCC(C=C4OC)=CC(OC)=C4OC	10.1016/S0928-0987(97)00020-1	1060	+2	0.902
**Patel1997-8**	C56H76N2O14	O=C(CCCC/C=C/C(OCCC[N@@+]([C@H](C(C=C1OC)=CC(OC)=C1OC)C2=CC(OC)=C3OC)(CCC2=C3)C)=O)OCCC[N@@+]([C@H](C(C=C4OC)=CC(OC)=C4OC)C(C5=C6)=CC(OC)=C6OC)(CC5)C	10.1016/S0928-0987(97)00020-1	1000	+2	2.363
**Patel1997-9**	C58H80N2O12S2	O=C(CC/C=C/CCC(SCCC[N@@+]([C@@H](C1=C2)CC(C=C3OC)=CC(OC)=C3OC)(CCC1=CC(OC)=C2OC)C)=O)SCCC[N@@+]([C@@H](C(C4=C5)=CC(OC)=C5OC)CC(C=C6OC)=CC(OC)=C6OC)(CC4)C	10.1016/S0928-0987(97)00020-1	1060	+2	3.741
**Patel1997-12**	C60H84N2O14	CC(COC(CC/C=C/CCC(OCC(C)C[N@@+]([C@@H](C1=C2)CC(C=C3OC)=CC(OC)=C3OC)(CCC1=CC(OC)=C2OC)C)=O)=O)C[N@+]4([C@@H](C(C(CC4)=C5)=CC(OC)=C5OC)CC(C=C6OC)=CC(OC)=C6OC)C	10.1016/S0928-0987(97)00020-1	1056	+2	3.434
**Clara2020-*m*-1a**	C24H34N4	C[N+]1(CCCC1)CC2=CC(/N=N/C3=CC(C[N+]4(C)CCCC4)=CC=C3)=CC=C2	10.1016/j.ejmech.2020.112403	378	+2	-2.202
**Clara2020-*m*-1b**	C26H38N4	C[N+]1(CCCCC1)CC2=CC(/N=N/C3=CC(C[N+]4(CCCCC4)C)=CC=C3)=CC=C2	10.1016/j.ejmech.2020.112403	406	+2	-1.084
**Clara2020-*m*-1c**	C28H42N4	C[N+]1(CCCCCC1)CC2=CC(/N=N/C3=CC(C[N+]4(CCCCCC4)C)=CC=C3)=CC=C2	10.1016/j.ejmech.2020.112403	434	+2	0.034
**Clara2020-*m*-1d**	C26H34N4	C[N+]1(CC=CCC1)CC2=CC(/N=N/C3=CC(C[N+]4(CC=CCC4)C)=CC=C3)=CC=C2	10.1016/j.ejmech.2020.112403	402	+2	-1.652
**Clara2020-*p*-1a**	C24H34N4	C[N+]1(CCCC1)CC2=CC=C(/N=N/C3=CC=C(C[N+]4(C)CCCC4)C=C3)C=C2	10.1016/j.ejmech.2020.112403	378	+2	-2.202
**Clara2020-*p*-1b**	C26H38N4	C[N+]1(CCCCC1)CC2=CC=C(/N=N/C3=CC=C(C[N+]4(CCCCC4)C)C=C3)C=C2	10.1016/j.ejmech.2020.112403	406	+2	-1.084
**Clara2020-*p*-1c**	C28H42N4	C[N+]1(CCCCCC1)CC2=CC=C(/N=N/C3=CC=C(C[N+]4(CCCCCC4)C)C=C3)C=C2	10.1016/j.ejmech.2020.112403	434	+2	0.034
**Clara2020-*p*-1d**	C26H34N4	C[N+]1(CC=CCC1)CC2=CC=C(/N=N/C3=CC=C(C[N+]4(CCC=CC4)C)C=C3)C=C2	10.1016/j.ejmech.2020.112403	402	+2	-1.652
**Clara2020-*m*-5b**	C19H24N3	C[N+]1(CCCCC1)CC2=CC=CC(/N=N/C3=CC=CC=C3)=C2	10.1016/j.ejmech.2020.112403	294	+2	1.383

**AV 391**	C54H71ClN2O15	C[N@+]1([C@@H](C(C=C2OC)=CC(OC)=C2OC)C(C(CC1)=C3)=C(OC)C(OC)=C3OC)CCCOC(/C(Cl)=C/C(OCCC[N@@+](CCC4=CC(OC)=C5OC)(C)[C@@H](C4=C5)CC(C=C6OC)=CC(OC)=C6OC)=O)=O	asaabstracts.com	1022	+2	1.863
**AV 069**	C54H71FN2O15	C[N@+]1([C@@H](C(C=C2OC)=CC(OC)=C2OC)C(C(CC1)=C3)=C(OC)C(OC)=C3OC)CCCOC(/C(F)=C/C(OCCC[N@@+](CCC4=CC(OC)=C5OC)(C)[C@@H](C4=C5)CC(C=C6OC)=CC(OC)=C6OC)=O)=O	asaabstracts.com	1006	+2	1.263
**NB 802-33**	C54H71ClN2O15	C[N@+]1([C@@H](C(C=C2OC)=CC(OC)=C2OC)C(C(CC1)=C3)=CC(OC)=C3OC)CCCOC(/C(Cl)=C/C(OCCC[N@@+](CCC4=CC(OC)=C5OC)(C)[C@@H](C4=C5OC)CC(C=C6OC)=CC(OC)=C6OC)=O)=O	asaabstracts.com	1022	+2	1.863
**AV 001**	C53H70N2O14	C[N@+]1([C@@H](C(C=C2OC)=CC(OC)=C2OC)C(C(CC1)=C3)=CC(OC)=C3OC)CCCOC(/C=C/C(OCCC[N@@+](CCC4=CC(OC)=C5OC)(C)[C@@H](C4=C5)CC(C=C6OC)=CC(OC)=C6OC)=O)=O	asaabstracts.com	958	+2	1.568
**AV 003**	C51H66N2O12	C[N@+]1([C@@H](C(C=C2OC)=CC=C2OC)C(C(CC1)=C3)=CC(OC)=C3OC)CCCOC(/C=C\C(OCCC[N@@+](CCC4=CC(OC)=C5OC)(C)[C@@H](C4=C5)CC(C=C6OC)=CC=C6OC)=O)=O	asaabstracts.com	898	+2	2.284
**NB 720-71**	C55H73ClN2O16	C[N@+]1([C@@H](C(C=C2OC)=CC(OC)=C2OC)C(C(CC1)=C3)=C(OC)C(OC)=C3OC)CCCOC(/C(Cl)=C\C(OCCC[N@@+](CCC4=CC(OC)=C5OC)(C)[C@@H](C4=C5OC)CC(C=C6OC)=CC(OC)=C6OC)=O)=O	asaabstracts.com	1052	+2	1.505

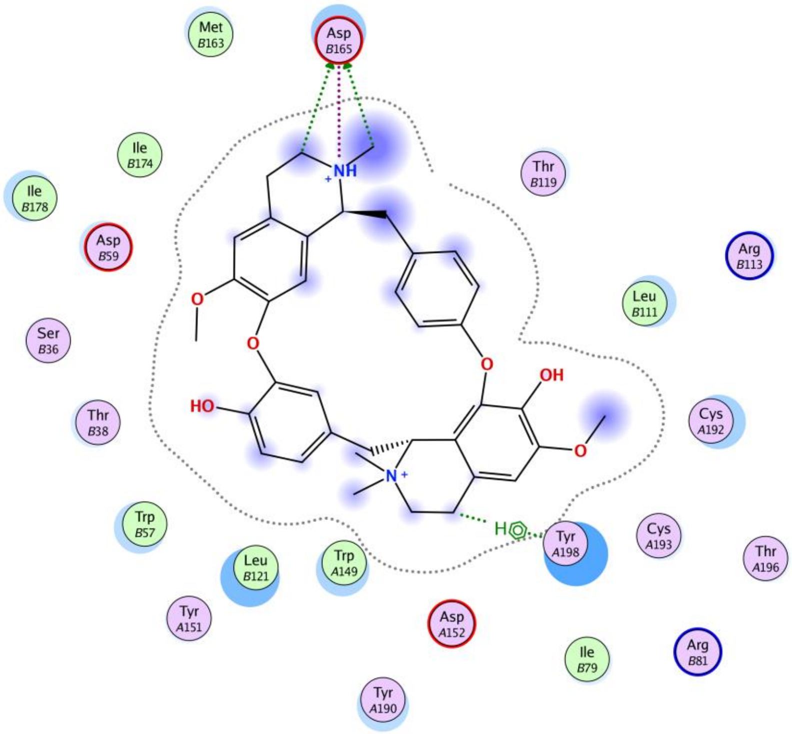

Figure S1. The binding modes of *d*-tubocurarine observed in the cryo-EM structure (PDB ID: 7SMS).
